# Neuromorphic device based on silicon nanosheets

**DOI:** 10.1038/s41467-022-32884-y

**Published:** 2022-09-05

**Authors:** Chenhao Wang, Xinyi Xu, Xiaodong Pi, Mark D. Butala, Wen Huang, Lei Yin, Wenbing Peng, Munir Ali, Srikrishna Chanakya Bodepudi, Xvsheng Qiao, Yang Xu, Wei Sun, Deren Yang

**Affiliations:** 1 State Key Laboratory of Silicon Materials & School of Materials Science and Engineering, Zhejiang University, 310027 Hangzhou, PR China; 2 ZJU-Hangzhou Global Scientific and Technological Innovation Centre, 310027 Hangzhou, PR China; 3 State Key Laboratory of Silicon Materials & School of Micro-Nanoelectronics, Zhejiang University, 310027 Hangzhou, PR China; 4 College of Information Science and Electronics Engineering, Zhejiang University, 310027 Hangzhou, PR China; 5 ZJU-UIUC Institute (ZJUI), Zhejiang University, 314400 Jiaxing, PR China; 6 New Energy Technology Engineering Laboratory of Jiangsu Provence & School of Science, Nanjing University of Posts and Telecommunications, 210023 Nanjing, PR China

**Keywords:** Electronic devices, Electronic devices

## Abstract

Silicon is vital for its high abundance, vast production, and perfect compatibility with the well-established CMOS processing industry. Recently, artificially stacked layered 2D structures have gained tremendous attention via fine-tuning properties for electronic devices. This article presents neuromorphic devices based on silicon nanosheets that are chemically exfoliated and surface-modified, enabling self-assembly into hierarchical stacking structures. The device functionality can be switched between a unipolar memristor and a feasibly reset-able synaptic device. The memory function of the device is based on the charge storage in the partially oxidized SiNS stacks followed by the discharge activated by the electric field at the Au-Si Schottky interface, as verified in both experimental and theoretical means. This work further inspired elegant neuromorphic computation models for digit recognition and noise filtration. Ultimately, it brings silicon - the most established semiconductor - back to the forefront for next-generation computations.

## Abstract (web summary)

Silicon is an abundant element on earth and is perfectly compatible with the well-established CMOS processing industry. Here, Sun et al. demonstrate multifunctional neuromorphic devices based on silicon nanosheet stacks, bringing silicon back as a potential material for neuromorphic devices.

## Introduction

Artificial neural network (ANN) is a new computation paradigm for big data concurrent processing and machine learning, which have been demonstrated as valuable tools for classification^1^, image processing^2,3^, and natural language processing^4^, and many other applications. However, massive concurrent calculations of ANN are challenges for traditional computational tools based on von Neumann architectures, limited by long latency for data transition from memories to arithmetic units. Therefore, some in-memory computation devices like memristors have been studied and exploited for ANN^5–8^. Although ANN is inspired by biological neural networks^9^, most architectures are not based on the exact same mechanism of the neurons. In the nervous system, the signals are event-driven discrete spikes, benefiting the high temporal information density while low energy consumption. Whereas traditional ANN signals transmission based on non-linear mapping of continuous values, for the sake of algorithms like backpropagation which are not explainable in biosystem. To inspect the functionality of the brain and inspire new calculation algorithms, spiking neuron network (SNN), a new type of ANN more closely mimicking the nervous system, has been received intense interest. SNN adopts discrete spike signals which carry temporal information including the time intervals and the rates^10^. As the nowadays developed calculation tools are not designed for discrete signals processing, novel synaptic devices and circuits need to be designed for hardware realization^11–14^. In this context, neuromorphic devices, including memristors and synaptic devices, which can well mimic some behavior of the nervous system, are believed crucial for next-generation computing^15–17^.

For neuromorphic computing, the simulation of biological learning patterns is the key target for its fundamental components. Unfortunately, this is hard to achieve by the Si and CMOS-based digital circuits. While many neuromorphic devices were developed based on organic semiconductors and quantum dots (QDs) and other emergence materials and new processing technology^18–20^. Moreover, the functioning mechanisms of these existing devices, including interface trap-induced neuromorphic behaviors, remained elusive and less controllable. Thus, it is crucial to investigate materials with neuromorphic behaviors and potential compatibility with Si technology, as well as to elucidate their underlying mechanism for further SNN applications. Recently, two-dimensional (2D) materials have come to the fore in materials science research, which demonstrate their superior fine-tuning electronic properties enabling the feasible device structure design^21–29^. Silicon nanosheets (SiNSs) are 2D thin films of Si which have different crystalline structures than bulk Si, while are potentially compatible with well-developed Si technology. Compared with bulk Si, SiNSs have exhibited distinct properties including large bandgap and quasi-direct bandgap, which potentially enable the fabrication of devices for different computation paradigm.

In this work, we demonstrated multifunctional neuromorphic devices based SiNS stacks. SiNSs were chemically exfoliated from CaSi_2_ in an ethanol solution of saturated hydrochloric acid with corresponding surface modification, enabling high quality for device fabrication. We found that the partially oxidized SiNS stacks had large capacitance and formed Schottky junctions with Au electrodes, which contributed to the charge storage and release and in turn the key functions in computational learning. Our devices exhibited the unipolar memristor characteristics and fast reset synaptic behavior without overcharge. Furthermore, the mechanism has been well investigated and verified by experimental, analytical, and numeric approaches. The revealed characteristics and mechanism of the devices have been implemented for pattern recognition and noise suppression by filtration, demonstrating the potential for device technologies and applications to next-generation neuromorphic computations.

## Results

### Design, synthesis, and characterization of the self-assembled 2D SiNS stacks

**Fig. 1:**
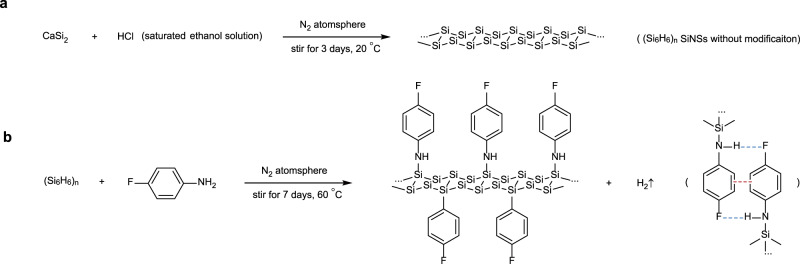
The reaction for preparation of SiNSs. **a** The pristine SiNSs **b** and further modification with p-fluoroaniline (pFA). Blue dashed lines correspond to hydrogen bonds and red dashed line corresponds to π–π interaction. H atoms bonded to Si are omitted for brevity.

We first prepared hydrogen (H)-terminated SiNSs by stripping Ca atoms off CaSi_2_ through topological exfoliation. To obtain high-quality SiNSs for device fabrication, intensive oxidation should be prevented. The H-terminated SiNSs prone to water-induced oxidation^30^. Therefore, instead of traditional water solution of hydrochloric acid^31^, the saturated ethanol solution (with a lower ionization equilibrium constant) was applied for the exfoliation in N_2_ atmosphere (Fig. 1a).

Further, p-fluoroaniline (pFA) was selected as the surface modifier. As a Lewis base, pFA reacted with the H-Si bond of H-terminated SiNSs (Fig. 1b)^32,33^. After the modification, energy dispersive spectrometer (EDS) mapping, X-ray photoelectron spectroscopy (XPS) (Fig. S1), and Fourier transform infrared (FTIR) spectroscopy, confirmed the absence of Ca and the successful attachment of pFA, (Fig. S2). The modification was designed and applied for multiple concerns: oxidation suppression, conductivity improvement, hierarchical stacking construction of SiNSs, and convenient solution processability. To be specific:

1. When capped by the pFA molecules, the steric hindrance mitigated the approach of oxygen and water molecules and thereby further oxidation^34^.
2. The electron-rich pFA increased the carrier density of the SiNSs and connected the SiNSs between stacks^35,36^. In particular, as the electron-donating group, the lone pairs of electrons of N atoms of pFA were shared with the SiNSs planes. Meanwhile, the π–π interaction (red dashed line in Fig. 1b) between pFAs enabled the electron hopping between layers, enhancing the conductance of the entire SiNS stacks.
3. Besides π-π interaction, hydrogen bond (blue dashed line in Fig. 1b) might also facilitate the self-assembly of SiNS stacks. The H atoms connected to N atoms were hydrogen-bond donors. And, the F molecules served as the hydrogen-bond acceptors (Fig. 1b). In addition, the pFA modifiers obstructed the direct contacts of SiNS layers and prevented aggregation of SiNSs.
4. The pFA modified SiNSs were readily dispersed in the pFA as a colloid for solution processing. After the evaporation of the dispersant pFA out of the dropped colloid, homogenous films could be formed on various nonpolar substrates, such as flexible Polyethylene terephthalate (PET), pristine silicon wafers without oxide, and silicon wafers with oxide but modified by a silane coupling agent (Fig. S3).

The obtained SiNSs enabled further device fabrication processes, including electrode patterning, material deposition, and other techniques potentially compatible with Si technology. Meanwhile, SiNS stacks possessed different characteristics than bulk Si. Spectroscopic and microscopic tools were applied before the further design of the devices.

**Fig. 2:**
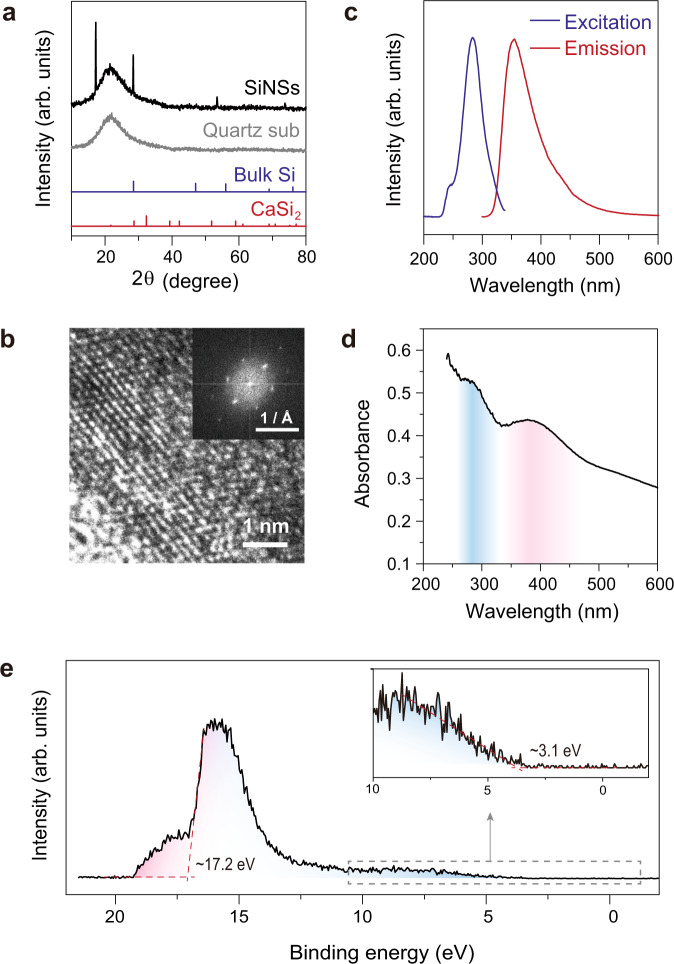
Material characterization. **a** X-ray diffraction (XRD) patterns of the as-synthesized silicon nanosheets (SiNSs) with p-fluoroaniline (pFA) modification, and the references (quartz substrate, bulk Si, CaSi_2_ powder). **b** High-resolution transmission electron microscopy (HRTEM) image of a SiNS showing lattice fringes, and the inset figure showing the corresponding image after Fast Fourier Transformation (FFT). **c** PL excitation (left) and emission spectra (right) of SiNSs. The emission spectrum was collected under 280 nm excitation, and the excitation spectrum was collected by using 350 nm as the emission wavelength. **d** Absorption spectrum of SiNSs, the blue and red shadows correspond to the resonance absorption peaks for excitation and emission, respectively. **e** Ultraviolet photoelectron spectrum (UPS) of SiNSs, the red shadow corresponds to high energy end for work function calculation and the blue shadow for Fermi level calculation.

XRD and HRTEM revealed the crystalline structure of SiNSs. The XRD patterns of the film exhibited sharp peaks. These peaks did not completely match the standard XRD patterns of neither bulk Si nor the starting material CaSi_2_, implying the formation of new crystalline phases. The sharp peak at 17.24° might correspond to the periodicity of modifier within the sheets, according to literature^37^. While, the position of one of the diffraction peaks of SiNS stacks (~28.4° with *d* = ~3.14 Å) was close to that of the (111) peak of Si (Fig. 2a). This might result from the similarity between the Si_6_ ring arrangement of SiNSs and (111) plane of bulk Si. Moreover, the HRTEM image also exhibited a graphene-like arrangement (Fig. 2b). A distance of ~1.80 Å was observed between the lattice fringes. Due to the tendency of forming staggered ABC stacks of SiNSs as found in other studies (Fig. S4)^38^, this observed distance between light spots corresponded with ~$1/\sqrt{3}$ of the interplanar distance.

Unlike bulk Si as an indirect bandgap semiconductor with poor response to light, the SiNSs instead exhibited intense photoluminescence (PL) peaks. The deep UV excitation band (~280 nm) and the emission band (~350 nm) were observed (Fig. 2c). The transient PL spectra monitored at 350 nm also revealed the short lifetime (~0.7 ns) of emission (Fig. S5, Table S1), which implied its quasi-direct bandgap nature. This was in contrast to the long PL lifetime of indirect bandgap Si usually observed (e.g., several tens of microseconds for silicon quantum dots^39^). Also, the UV–Vis absorption spectrum showed the resonance absorption enhancement at the same wavelengths of PL peaks (Fig. 2d). Estimated from the PL emission peak and Tauc plot of absorption spectrum (Fig. S6), the wide bandgap energy of SiNSs was calculated to be around 3.2–3.5 eV, consistent with similar materials reported in other studies^32,33^.

We also inspected the energy band properties of SiNS stacks, which are crucial for device fabrication and analysis. From a Hall measurement, the *n*-type conductivity of SiNSs and high carrier density (~2.5 × 10^15 ^cm^−3^) were revealed (Table S2). Without traditional group 5 elements (like P) as donors, the good *n*-type conductivities resulted from the electron doping effect of N atoms of pFA modifiers. Comparably high mobility (~300 cm^2^ V^−1^ S^−1^ Table S2) was observed, which is one magnitude higher than the value formerly reported for single-layer silicene^40^. The work function and Fermi level were estimated by ultraviolet photoelectron spectroscopy (UPS) (Fig. 2e). Due to the inevitable partial oxidation, a broad peak of insulating oxide was also observed at the high binding energy side. Extending the photoemission cut-off, the work function was calculated to be ~4.06 eV. Besides, according to the low binding energy side, the Fermi level was ~3.10 eV above the valence band edge, which confirmed the *n*-type conductivity.

### The coupling of capacitance and Schottky junction based on 2D SiNS stacks and induced memristor behavior of devices

**Fig. 3:**
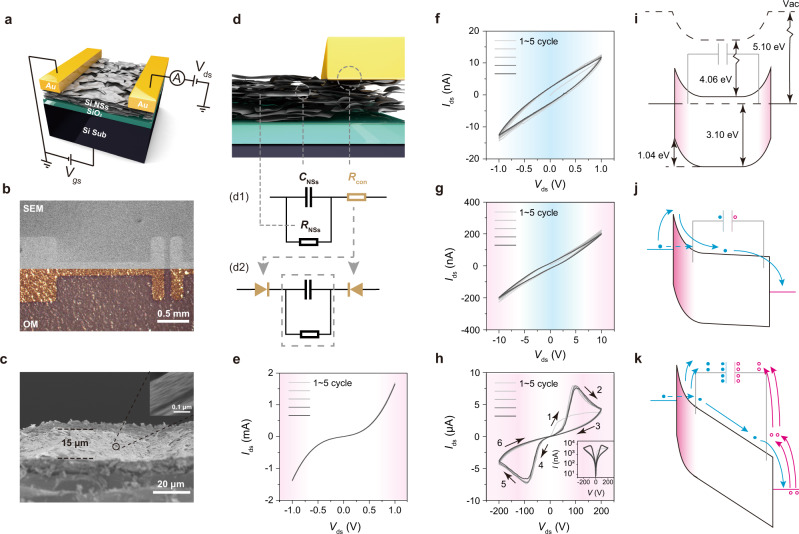
Memristor behavior and mechanism. **a** Schematic figure of the device made of SiNSs. **b** Scanning electron microscopy (SEM) and optical microscopic images of the device. **c** Cross-section SEM image of the active SiNS layer of a device. **d** Schematic figure illustrating the proposed models of the device. (d1 for the constant contact resistance model and d2 for the varying contact resistance model) The quasi-static *I–V* sweep loop of the device **e** with good annealing under 1 V, without good annealing under **f** 1 V, **g** 10 V, **h** 200 V, the blue shadow regions comply with the constant contact resistance model, and the red shadow regions correspond to the varying contact resistance. **i** The band diagram of the device at the equilibrium state. The band diagram of the transformation of devices from **j** original state to **k** low resistive state. The blue dots correspond to electrons, while the red circles correspond to holes. The red region of the band diagram corresponds to the depletion region.

Based on the energy band and stacking structure of the SiNSs, we designed two-terminal devices (Fig. 3a, b), that possessed intriguing hysteretic characteristics and memristor-like behavior. The mechanism behind the devices was different from common memristors that depend on conductive filaments or ion migration, or other structures^5^. The characteristics of our devices were instead induced by the coupling of the large capacitance of the partially oxidized SiNS stacks and the Schottky junctions between the electrodes and SiNSs stacks. To figure out the underlying physics, a simple model was proposed (Fig. 3d1). In the model, *C*_NSs_ corresponds to the capacitance of partially oxidized SiNSs stacks and *R*_con_ corresponds to the contact resistance. In addition, we have included *R*_NSs_ in the model, which corresponds to the gross resistance in the stacks.

The large capacitance mainly resulted from the hierarchical microscopic structure of the partially oxidized SiNS stacks. The thickness of the SiNS film was ~15 μm within an optimal range (Fig. 3c and Fig. S7). Sheets were assembled into bundles, which in turn were assembled into stacks. The thickness of the bundles was ~20 nm from the inset in Fig. 3c. All of these structures collectively contributed to capacitance, i.e., the transfer of carriers can be hindered by the gaps and the partial oxidation between lamellar structures, which can be regarded as tiny plate capacitors.

Moreover, Au was chosen as the electrodes instead of forming an Ohmic contact. The work function of Au is comparatively high (5.1 eV), which resulted in two opposite Schottky junctions between Au and SiNSs with high barriers of ~1.04 eV (Fig. 3i). Besides, as Au is stable and inert, it is less prone to form conductive filaments and thus avoids accompanying disruptive behaviors.

The model was verified by consecutive experiments. First, a sufficient heat treatment (200 °C for 30 min in an N_2_ filled glove box) was applied, which thermodynamically and kinetically facilitated rearrangement, thus a better contact between SiNS layers with the stacking structure retained (Fig. S8). Therefore, less oxidation and higher conductivity of SiNS stacks were achieved (Fig. S9). In this case, the small *R*_NSs_ short-circuited *C*_NSs_ (shown in the dashed line box, Fig. 2d). Therefore, symmetric *I–V* curves without hysteresis loops were observed (Fig. 3e), which corresponded to the typical pattern of back-to-back Schottky junctions.

In contrast, different characteristics were observed if the devices were annealed to a less extent (100 °C for 10 min in a glove box). Smaller current signals and larger *R*_NSs_ and *R*_con_ were observed (Fig. 3f), which resulted from the partial oxidization of SiNS stacks. The hysteresis loops were observed, due to the large *C*_NS_. When the applied scanning voltage was within 1 V, *R*_con_ was assumed as a constant value due to the low voltage across junctions (Fig. 3d1). Using the constant *R*_con_ model, the output current followed Eqs. (1) and (2), which well described the experimental result:

1 $${i}_{{{{{{\rm{ds}}}}}}}\left({v}_{{{{{{\rm{ds}}}}}}}\right)=\frac{{V}_{0}{C}_{{{{{{\rm{NSs}}}}}}}{R}_{{{{{{\rm{NSs}}}}}}}{R}_{{{{{{\rm{con}}}}}}}}{{{t}_{0}\left({R}_{{{{{{\rm{NSs}}}}}}}+{R}_{{{{{{\rm{con}}}}}}}\right)}^{2}}\left(1-{{{{{{\rm{e}}}}}}}^{-\frac{{R}_{{{{{{\rm{NSs}}}}}}}+{R}_{{{{{{\rm{con}}}}}}}}{{C}_{{{{{{\rm{NSs}}}}}}}{{R}_{{{{{{\rm{con}}}}}}}}^{2}}\,\frac{{t}_{0}}{{V}_{0}}\,{v}_{{{{{{\rm{ds}}}}}}}}\right)+\frac{{v}_{{{{{{\rm{ds}}}}}}}}{{R}_{{{{{{\rm{NSs}}}}}}}+{R}_{{{{{{\rm{con}}}}}}}}+{i}_{0}\;\;\;\;\;({{{{{\rm{upper}}}}}}\;{{{{{\rm{curve}}}}}})$$

2 $${i}_{{{{{{\rm{ds}}}}}}}\left({v}_{{{{{{\rm{ds}}}}}}}\right)=\frac{{V}_{0}{C}_{{{{{{\rm{NSs}}}}}}}{R}_{{{{{{\rm{NSs}}}}}}}{R}_{{{{{{\rm{con}}}}}}}}{{{t}_{0}\left({R}_{{{{{{\rm{NSs}}}}}}}+{R}_{{{{{{\rm{con}}}}}}}\right)}^{2}}\left({{{{{{\rm{e}}}}}}}^{-\frac{{R}_{{{{{{\rm{NSs}}}}}}}+{R}_{{{{{{\rm{con}}}}}}}}{{C}_{{{{{{\rm{NSs}}}}}}}{{R}_{{{{{{\rm{con}}}}}}}}^{2}}\ \frac{{t}_{0}}{{V}_{0}}\,{v}_{{{{{{\rm{ds}}}}}}}}-1\right)+\frac{{v}_{{{{{{\rm{ds}}}}}}}}{{R}_{{{{{{\rm{NSs}}}}}}}+{R}_{{{{{{\rm{con}}}}}}}}+{i}_{0}^{{\prime} }\;\;\;\;\;({{{{{\rm{lower}}}}}}\; {{{{{\rm{curve}}}}}})$$

where *i*_ds_ was the channel current, *v*_ds_ was the applied voltage, and *i*_0_ was the small deviation of the leakage current from *C*_NSs_.

The derivation can be found in Fig. S10. Using this equation for fitting, the *C*_NSs_, *R*_NSs_, *R*_con_ were extrapolated to be ~7 × 10^−7 ^F, ~1 × 10^9^ Ω, and ~6 × 10^7^ Ω (Fig. S11 and Table S3). Additionally, considering the physical dimensions of the device and SiNSs, the capacitance was estimated as ~1 × 10^−7 ^F, which was close to the extrapolated result found from fitting.

After confirming the model, we further explored memristor-like behavior. When we increased the range of scanning voltage, the *I–V* curves gradually deviated from Eqs. (1) and (2) (Fig. 3g). The deviation was due to the varying contact resistance (*r*_con_) of the Schottky contacts (Fig. 3d2). The larger voltage across the Schottky contacts caused the breakdown and the decrease of *r*_con_. When further increasing the applied voltage, the significantly increased avalanche (or tunneling) current also caused the negative differential resistance (NDR) effect of *r*_con_^41^.

Taking advantage of the NDR and the capacitance of SiNS stacks, the unipolar memristor-like behavior was achieved (Fig. 3h), as the resistance of the device was controlled by the value other than the direction of the voltage. Unlike the common memristors, which begin in the high resistive state (HRS) and then transform to the low resistive state (LRS), our devices instead showed the LRS first. When the increasing applied voltage caused the NDR of the Schottky junction, the voltage of junction (*v*_con_) suddenly decreased while the current (*i*_ds_) still increased. Therefore, the voltage (*v*_NSs_ = *v*_ds_−*v*_con_) across SiNSs (also *C*_NSs_) suddenly increased. Then, the quickly increasing charging current of *C*_NSs_ was observed, which corresponded to the LRS (Fig. 3j, k). When the *C*_NSs_ was gradually charged, the current of the device then decreased quasi-exponentially, as per Eq. (2), and the device shifted to the HRS. After this, the device remained as HRS, when the applied voltage decreased to zero. That resulted in the charges in the *C*_NSs_ that reduced the Schottky barrier height (Fig. 3k), and the breakdown and NDR effect could not take place. The difference in current between the LRS and HRS was over one order of magnitude, which promised the differentiation between these two states (Fig. 3h inset). When flipping the direction of the applied voltage, the same behavior was observed, which corresponded to the unipolar behavior of the device. The overall process only involved reversible charge/discharge of the *C*_NSs_ and the breakdown/recovery of the Schottky junction (*r*_NSs_) without structural changes. Therefore, the *I–V* curves of the consecutive cycling tests overlapped well (Fig. 3h).

Also, the mechanism of the devices proved to be universal, which was verified by replacing the Si substrate with PET (Fig. S12) and reproducing the *I–V* curve by commercial electronic components (Fig. S13). Meanwhile, some non-idealities, like the circle-to-circle coherency still require improvement (the first circle was different from others, Fig. S14).

### Synaptic behavior for the spiking neural networks

**Fig. 4:**
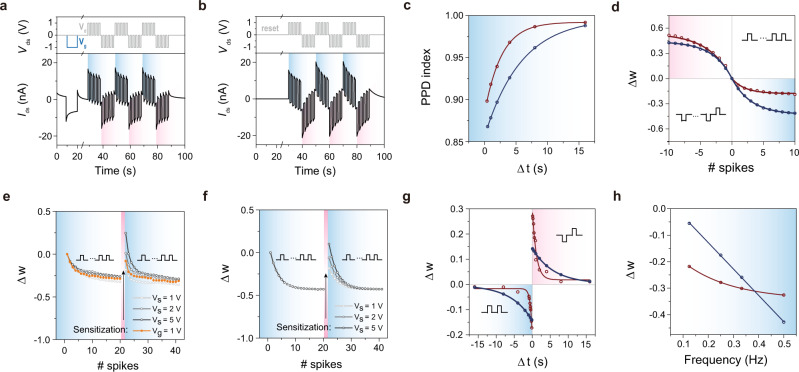
Experimental and numeric simulation results of the synaptic behavior of the devices. **a** The experimental result and **b** the simulation result of depression and restoration of the synaptic devices. **c** Paired-pulse depression (PPD). **d** Long-term memory (LTM), where the red line is the experimental result and the blue line is the simulation result. **e** The experimental result and **f** the simulation result of non-associative learning (NAL) behavior of the devices. **g** Spike timing-dependent plasticity (STDP), and **h** spike rate-dependent plasticity (SRDP) of devices, where the red line is the experimental result and the blue line is the simulation result. In figures, the blue shadow regions correspond to the inhibition and red shadow regions for excitation.

Next, based on the revealed mechanism, we further investigated the synaptic behavior of the device, by applying consecutive voltage spikes (Fig. 4). The applied spike voltage was 1 V to ensure low energy consumption without the NDR effect (Fig. S15). Also, using the previously extrapolated values of *C*_NSs_, *R*_NSs_, and *R*_con_ with some modification (Fig. S16), numerical simulations were conducted for comparison. In this work, we mainly focused on the proof of concept and mechanism, and we are still working on improving the device performance (Fig. S17).

Subjected to spikes of the same direction, the synaptic weight continuously decreased. The originally large synaptic weight was due to the charging current of *C*_NSs_. After continuous excitations, *C*_NSs_ was gradually charged, which led to the lower current and depression (Fig. 4a, b). During the interval without spikes, only small leaky currents were found (Fig. S18), due to the large *R*_con_ and long lifetime of stored carriers. The paired-pulse depression (PPD) of our devices was thus found, as the two successive spikes were applied. Also, for the leakage, the PPD index increased when the interval period lengthened (Fig. 4c). Moreover, excited by the spikes of the opposite polarity, the current enhanced in that opposite polarity, and the synaptic weight reset immediately. The restoration was due to the depletion of carriers in the *C*_NSs_ under negative bias (Fig. 4a, b).

Furthermore, we experimentally and theoretically evaluated long-term memory (LTM) of the devices. We plotted the synaptic weight versus the number of spikes. The positive excitation number denotes the spikes of the same direction, and the negative one denoted that of the opposite polarity corresponding to the final inspected spike (Fig. 4d). From the experimental result, the synaptic weight change agrees with a quasi-exponential pattern versus increasing numbers of applied spikes. Using the constant *R*_con_ model (Fig. 3d1), the analytical synaptic weight followed Eq. (3) (the detailed derivation is in Fig. S19).

3 $$\triangle w\left(n\right)=\frac{{i}_{{{{{{\rm{dsn}}}}}}}-{i}_{{{{{{\rm{ds}}}}}}0}}{{i}_{{{{{{\rm{ds}}}}}}0}}=\left\{\begin{array}{c}{A}_{1}\left(1-{{{{{{\rm{e}}}}}}}\,^{\frac{n}{\frac{{C}_{{{{{{\rm{NSs}}}}}}}{{R}_{{{{{{\rm{con}}}}}}}}^{2}}{2{t}_{{{{{{\rm{spk}}}}}}}\left({R}_{{{{{{\rm{NSs}}}}}}}+{R}_{{{{{{\rm{con}}}}}}}\right)}}}\right)\,\;\;\;\;\;(n\, > \, 0)\\ -{A}_{1}\left(1-{{{{{{\rm{e}}}}}}}^{\frac{n}{\frac{{C}_{{{{{{\rm{NSs}}}}}}}{{R}_{{{{{{\rm{con}}}}}}}}^{2}}{2{t}_{{{{{{\rm{spk}}}}}}}\left({R}_{{{{{{\rm{NSs}}}}}}}+{R}_{{{{{{\rm{con}}}}}}}\right)}}}\right)\;\;\;\;\;(n\, < \,0)\end{array}\right.$$

where *i*_ds0_ was the device current without former spikes, *i*_dsn_ was the device current excited by n former spikes. A_1_ was a constant value relevant to *C*_NSs_, *R*_NSs_, *R*_con_, and applied spike voltage (*V*_spk_ = 1 V, *t*_spk_ = 1 s). The simulation results agreed with the analytical expression and qualitatively agrees with the experiment results. Note that the experimental curve was not completely symmetric about the original point. It was due to the impedance of the Schottky barrier varied by the direction of the last spike. The deviations from the experimental curves could be attributed to the actually non-constant nature of *r*_con_ discussed previously, i.e., when the last spike was of opposite polarity, the Schottky barrier was built up, leading to a smaller resistance.

Benefiting from LTM, our devices can mimic non-associative learning (NAL), an important behavior for biological adaptation to external stimuli^42^. The two primary patterns of NAL are habituation and sensitization. Habituation corresponds to the diminution of the response after continuous exposure to repetitive stimuli. Sensitization corresponds to the enhancement of the response upon a new different stimulus. LTM of consecutive spikes mimicked habituation, whereas large voltage excitation of the opposite polarity reset the device. Especially, the first 1 or 2 points could be sensitized and higher than the original value, and then followed by normal habituation again. The larger the applied opposite voltage, the more significant enhancement of the first several spikes. The synaptic weight after opposite spikes could be even higher than the original state (Fig. 4e), as the opposite spike depleted the charges and then recharged the device in the opposite direction. The simulation also revealed the same phenomenon (Fig. 4f).

For synaptic devices, immediate restoration without over-compensation is important. For our devices, a complete reset could be easily achieved by tuning the energy band structure. Besides negative voltage spikes on the drain, the sensitization and complete reset could be achieved when a positive voltage spike was applied on the back gate (the heavily doped silicon wafer substrate) (Fig. 4e orange line). It added up the voltage across the depletion region of Schottky junctions. A comparably low back gate voltage (*V*_g_ = 1 V) was enough to facilitate the tunneling current of the Schottky junction and depletion of the carriers in the *C*_NSs_, and thus sensitization (Fig. S20). Different from the negative spike applied on the drain, it eliminated the recharging of the opposite carriers and over-sensitization. Therefore, for the measurements, we used this method to reset devices.

Spike timing-dependent plasticity (STDP)^43^ was also demonstrated. The depression or potentiation was induced by two aligned or opposing direction pulses, respectively. Also using the constant *R*_con_ model, the synaptic weight changed exponentially when varying the interval between spikes (Δ*t*) (Fig. S21), as shown in Eq. (4).

4 $$\triangle w\left(\triangle t\right)=\frac{{i}_{{{{{{\rm{ds}}}}}}}\left({2t}_{{{{{{\rm{spk}}}}}}}+\triangle t\right)-{i}_{{{{{{\rm{ds}}}}}}0}}{{i}_{{{{{{\rm{ds}}}}}}0}}=\left\{\begin{array}{c}{A}_{2}{{{{{{\rm{e}}}}}}}^{-\frac{\triangle t}{\frac{{C}_{{{{{{\rm{NSs}}}}}}}{{R}_{{{{{{\rm{con}}}}}}}}^{2}}{{R}_{{{{{{\rm{NSs}}}}}}}+{R}_{{{{{{\rm{con}}}}}}}}}}\;\;\;({{{{{{\rm{opposite}}}}}}}\;{{{{{{\rm{direction}}}}}}})\\ -{A}_{2}{{{{{{\rm{e}}}}}}}^{-\frac{\triangle t}{\frac{{C}_{{{{{{\rm{NSs}}}}}}}{{R}_{{{{{{\rm{con}}}}}}}}^{2}}{{R}_{{{{{{\rm{NSs}}}}}}}+{R}_{{{{{{\rm{con}}}}}}}}}}\;\;\;\;\;({{{{{{\rm{same}}}}}}}\;{{{{{{\rm{direction}}}}}}})\end{array}\right.$$

where *i*_ds_(2*t*_spk_ + Δt) was the current after two coupling spikes and A_2_ was another constant value that was also relevant to *C*_NSs_, *R*_NSs_, *R*_con_, *V*_spk_ = 1 V and *t*_spk_ = 1 s.

The STDP of the synaptic devices could be exploited in SNNs, as they map the temporal information to weight changes. A similar asymmetry of the experimental curve was due to the same reasoning given for LTM. Meanwhile, a larger discrepancy between the experimental result and simulation was found for STDP (Fig. 4g). This was because the capacitance of *C*_NSs_ varied under different excitation frequencies. For the STDP case, the interval between spikes could be as small as 0.1 s, which resulted in smaller *C*_NSs_. Comparing the experimental and simulation result of spike rate-dependent plasticity (SRDP), the deviation from the simulation was also found (Fig. 4h).

### Spiking neural network simulations

Our devices had a similar STDP pattern as biologic synapses and thus were used to build an SNN to investigate the potential for unsupervised neuromorphic computing to deal with samples without labels. This brian-inspired STDP algorithm was based on the automatic change of synapse weights when subjected to different stimuli or inputs. When the synapse-connected two neurons are firing sequentially, the weight of the connected synapse increases according to the interval between the two fires. The shorter the interval, the larger the weight change. Then, after many stimuli, the network can make better and better responses.

**Fig. 5:**
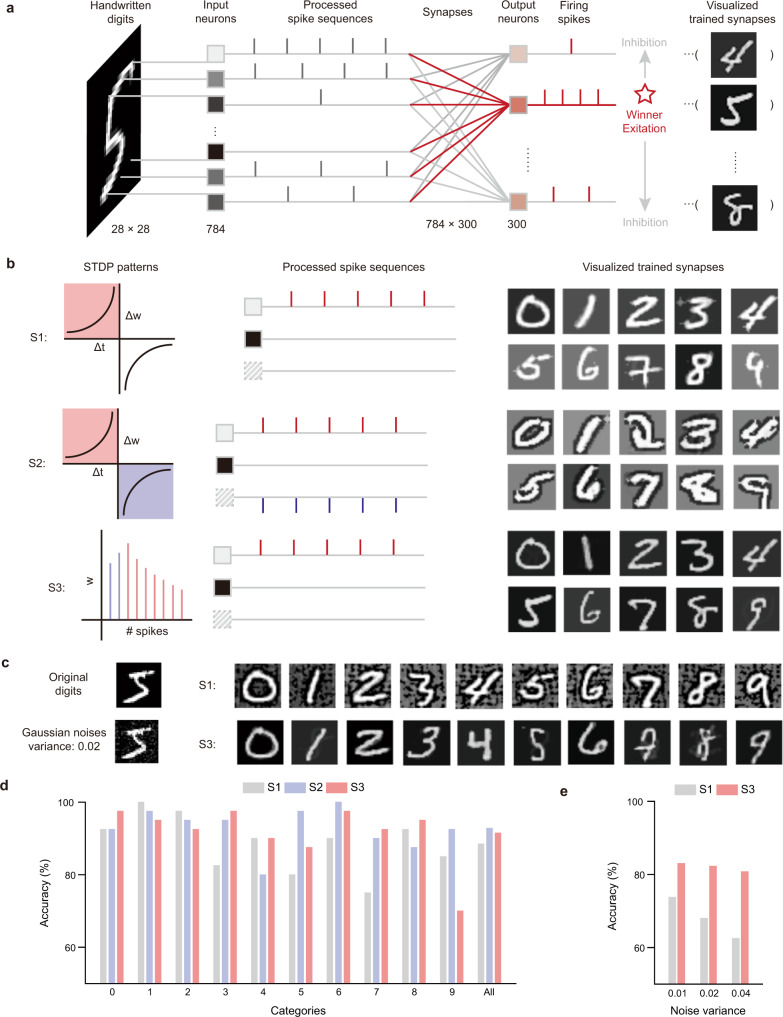
Spiking neural network simulations. **a** Spiking neuron network (SNN) architecture. **b** Several visualized synapses for simulations S1, S2, and S3. **c** Visualized synapses of a noise test for S1 and S3. **d** Recognition accuracies in different categories for three simulations S1, S2, and S3. **e** Recognition accuracies of noises test for S1 and S3.

To illustrate the potential for neuromorphic computing, we did a simple proof-of-concept demonstration using a small set of actual devices (Fig. S22), which demonstrated the potential classification application of the device-comprising neuron network. Furthermore, to explore the theoretical potential, we carried out simulation experiments based on the device STDP for a more complicated task, i.e. the Modified National Institute of Standards and Technology database (MNIST) handwritten digits^44^ recognition based on an unsupervised SNN (Fig. 5a)^45^. Three different simulations were implemented and compared based on the characteristic of the device: only potentiation input encoding, both potentiation and depression input encoding, and the abnormal NDR effect induced by high voltage input spikes, respectively. To be more specific, the first simulation (S1) encoded only potentiation spike sequences, which corresponded to the second quadrant of the STDP pattern, (Fig. 4g). Meanwhile, the second simulation (S2) considered both the potentiation and depression STDP pattern (second and fourth quadrants in Fig. 4g). Therefore, we expected S2 would have higher accuracy than S1. Then for the third simulation (S3), we investigated the special STDP pattern found for devices under large spike voltage while with the same encoding method as S1. Due to the NDR, the device synaptic weight first exhibited an abnormal inverse pattern (Fig. S15d). Compared to S1, anti-noise properties were expected for S3 as the low level of grayscale noise could be filtered by the first inverse spikes.

Some of the trained synapses were visualized in Fig. 4b to demonstrate the learning efficiency of different simulations. Comparing the visualized synapses of bi-directional S2 to simplest S1, an apparent edge effect was found due to the bi-directional STDP encoding (Fig. 5b). The edge increased the contrast between the pattern and background, which improved the recognition accuracy. Furthermore, the abnormal-inverse S3 was compared to S1. Even though the encoding strategy is the same, the visualized synapses of S3 appear “darker” than that of S1 (Fig. 5b), as the stimulus should be stronger to strengthen the synapse for the first inverse spikes. Therefore, higher accuracy of S2 over S1 or S3 could be expected. To validate the anti-noise properties, images with Gaussian noises were further applied as training sets (Fig. S23b). Obviously, compared with S1, the visualized synapses of S3 were found to filter more noises and have greater fidelity to the original digits, which verified the anti-noise ability of S3 over S1(Fig. 5c).

Finally, we compared the recognition accuracy and anti-noise capabilities of three simulations. The overall accuracies to identify the original MNIST handwritten digits without additional noise were nearly 90%, (in all cases 88.50%, 92.80%, and 91.50% for S1 through S3, respectively Fig. 5d). For the accuracies of individual digit categories, we found that S2 had a higher average recognition accuracy (> 80% for each number). In comparison, S1 and S3 had the lowest accuracy of 75% for the digit “7” and 70% for the digit “9”, respectively. In addition, S3 had higher recognition accuracy when confronted with noises. When the noise level increased (Gaussian noise with variances of 0.01, 0.02, and 0.04), the accuracy of S1 decreased rapidly while that of S3 reduced only slightly (Fig. 5e).

## Discussion

This work demonstrated the versatile SiNSs-based neuromorphic device, which extended Si technology to next-generation computation like SNN, a crucial step towards cognitive integration in artificial intelligence systems. We investigated the unipolar memristor-like behavior based on the NDR and device capacitance, as well as the synaptic response with effective reset ability based on the high capacitance originated from the layered structure and the rectification behavior of SiNSs-Au junctions. Then, we analytically described characteristics, including LTM, STDP, which were essential for neuromorphic calculations. Finally, we demonstrated an SNN inspired by device behaviors, which was proved effective for digit recognition and noise filtration.

In addition, we envision other emerging research areas to encourage in-depth investigation for the potential of SiNSs in SNN and their integration with Si-based electronics. First, photoemission of SiNSs demonstrated the possibility for optoelectronic. Second, given the compatibility with the solution process on soft substrate like PET, our SiNSs demonstrate the possiblility for flexible device fabrication^46,47^. Third, the CMOS-compatible SNN arrays are attractive for fabrication with existing Si technology. These exciting topics require further study to bridge the gap between technologies of artificial intelligence and the well-established Si industry.

## Methods

### Material preparation

The ethanol solution of saturated hydrochloric acid was prepared by using 50 mL ethanol (Sinopharm Chemical Reagent Co. Ltd) to absorb the bubbled-through hydrogen chloride gas generated by mixing 10 g sodium chloride with 10 mL 98% sulfuric acid.

For obtaining SiNSs, about 0.5 g of CaSi_2_ crystallites (Sigma-Aldrich) was immersed in the ethanol solution of saturated hydrochloric acid (50 mL) in a Schlenk flask. The mixture was stirred continuously for 3 days under the N_2_ atmosphere to obtain the H-terminated SiNSs without modification. The mixture was then filtered in the Schlenk line. The filter residue was mixed with 50 mL p-fluoroaniline (pFA) (Macklin) also in a Schlenk flask. The mixture was then stirred continuously for 7 days at room temperature under the N_2_ atmosphere to obtain the modified SiNSs. The mixture was then collected and stored in a glove box filled with N_2_.

### Device fabrication

The heavily p-doped <100> silicon wafer (ρ < 0.02 Ω cm) with a 200 nm SiO_2_ field oxide layer was chosen as the substrate and cut into 1 × 1 cm^2^. The surface of the wafers was functionalized to be nonpolar by immersion in a toluene solution of (3-aminopropyl) trimethoxysilane (1.25 mL mL^−1^) (Macklin) at 60 °C for 4 min in a glove box of N_2_ atmosphere. Excessive molecules were removed by rinsing and sonicating in toluene, and isopropanol. These substrates were then dried and collected. The active layer of pFA modified SiNSs was formed by dropping 80 μL mixture of and SiNSs and pFA onto the wafer in a glove box of N_2_ gas. The solvent of pFA was evaporated, and the active layer was further thermally treated on a hot plate at 100 °C for 10 min or 200 °C for 30 min to achieve moderate or sufficient annealing in a glove box. Further, 120 nm thick gold electrode was then thermally evaporated on the active SiNSs layer via a shadow mask, leading to 100 μm long and 1000 μm wide channels.

### Characterization

The X-ray diffraction (XRD) patterns were obtained by using a SHIMADZU LabX XRD6000 diffractometer with Cu Kα (λ = 1.5406 Å) radiation. The JEM 2100F transmission electron microscope with an acceleration voltage of 200 kV was used to obtain TEM images. The PL system (F920, Edinburgh Instruments) was used for collecting PL. During the transient PL measurements, a 280 nm wavelength pulsed excitation source with a frequency of 50 Hz (µF920H) was used. The optical absorption was obtained from a UV–vis–NIR spectrometer (HITACHI U-4100). X-ray photoelectron spectroscopy (XPS) measurements were performed by an x-ray photoelectron spectrometer (Kratos AXIS Ultra DLD). UPS measurements were performed by using Thermo Scientific ESCALAB 250Xi with a 21.2 eV He-Ia source. FTIR spectrometer (JASCO FT/IR-6100) operated in the transmission mode with a resolution of 4 cm^−1^ was used to measure all the samples. The hall measurement was conducted on a Lakeshore 7604. The cross-section SEM images of a typical device were obtained by using a scanning electron microscope (HITACHI S4800) at an acceleration voltage of 5 kV. The device performances were measured by the semiconductor parameter analyzer (FS480, PDA Co. Ltd). For the quasi-static *I–V* scanning, the time interval between two sample points was 0.04 s, and between 0 and maximum applied voltage, there were 100 evenly distributed sample points. For I-t scanning, the time interval between the sample points was also 0.04 s, and the time for voltage spike (1 V) was 1 s.

### Simulation

The quasi-static *I–V* curve and synaptic behavior of devices were simulated by using the model of capacitors and resistors on Simulink (Matlab R2020a). The value of the resistors was extracted in the quasi-static *I–V* measurement: *R*_NSs_, *R*_con_ were set as ~1 × 10^9^ Ω and ~6 × 10^7^ Ω, respectively, whereas the *C*_NSs_ was modified to be 1 × 10^−7 ^F for better agreement with the experiment result.

### SNN training

The SNN consisted of 2 layers with 784 input neurons and 300 output neurons, respectively (Fig. 5a). The 784 × 300 synapses connected those two layers. First, the images with 28 × 28 (784) pixels were processed by a convolution with a receptive-field kernel (Fig. S23a) to generate corresponded spike sequences by input neurons according to grayscale^48^. Then, the output neurons were updated subjected to programmed input spikes following the STDP models of our devices in order to represent different digital categories. Three different simulations were carried out with different input encoding. For S1, the spike frequency was proportional to the grayscale of the corresponding pixel, and no spikes were applied for grayscale ≤ 0. For S2, the grayscale higher than 0 was encoded with potentiation spike sequences while lower than 0 was encoded with depression spike sequences. For S3, the encoding strategy was similar to S1 but showed an inverse effect in the first several input spikes. Finally, according to the leaky-and-integrate-fire (LIF) model^49^ and winner-take-all strategy, the output neuron with the most firing spikes was selected to give the recognition result. The SNN training in our simulations mainly consisted of two steps. One was the synapses weights updating and the other one was categories labeling. For the weights updating step, the membrane potential of output neurons are accumulated according to the input spikes and the weights from their corresponding synapses. At the time if the potential of one output neuron exceeded the threshold and fires, the connected synapses who translated input spikes that contributed to the firing behaviors will be strengthened, in which case the changes of the weights were proportional to the interval between firing time and the pre-synapse spiking time given by the STDP model in Fig. 5b. Instead, synapses that didn’t contribute to the firing neuron will be weakened. Besides, the variable threshold and lateral inhabitation were also considered which means if an output neuron fired, its threshold would be increased to keep the homeostasis among other neurons and it would also lower down the membrane potential of other output neurons. In the category labeling step, fixing the threshold and the trained weights, the training set is presented to the trained network again and the output neurons are labeled to the patterns categories according to its most firing times to the corresponding input image^2^. Furthermore, the decay values of membrane potential and the threshold for each neuron are 0.8 and 0.4, relatively.

## Supplementary information

Supplementary Information

## Data Availability

All figure data supporting this study are available within the article and its Supplementary Information. The raw figure data generated in this study have been deposited in the Figshare database under accession code [10.6084/m9.figshare.20436558].

## Code availability

The codes used for the simulations are described in [https://github.com/xuxinyigege/SNN-demo.git] or are available from the corresponding author upon reasonable request.

## References

[1] Janocha K, Czarnecki WM (2016). On loss functions for deep neural networks in classification. Scheda. Inform..

[2] Xu H, Ma J, Zhang XP (2020). MEF-GAN: Multi-Exposure Image Fusion via Generative Adversarial Networks. IEEE Trans. Image Process.

[3] Xu, H., Liang, P., Yu, W., Jiang, J. & Ma, J. Learning a generative model for fusing infrared and visible images via conditional generative adversarial network with dual discriminators. In: *IJCAI'19: Proc. 28th International Joint Conference on Artificial Intelligence.*10.24963/ijcai.2019/549 (2019).

[4] Liu W (2017). A survey of deep neural network architectures and their applications. Neurocomputing.

[5] Jeong DS, Hwang CS (2018). Nonvolatile memory materials for neuromorphic intelligent machines. Adv. Mater..

[6] Choi C (2020). Curved neuromorphic image sensor array using a MoS2-organic heterostructure inspired by the human visual recognition system. Nat. Commun..

[7] Chen S, Lou Z, Chen D, Shen G (2018). An artificial flexible visual memory system based on an UV-motivated memristor. Adv. Mater..

[8] Huang W (2021). Memristive artificial synapses for neuromorphic computing. Nano-Micro Lett..

[9] Slavík J, Čmiel V, Hubálek J, Yang Y, Ren TL (2021). Hippocampal neurons’ alignment on quartz grooves and parylene cues on quartz substrate. Appl. Sci..

[10] Tavanaei A, Ghodrati M, Kheradpisheh SR, Masquelier T, Maida A (2019). Deep learning in spiking neural networks. Neural Netw..

[11] Wang Z (2020). Toward a generalized Bienenstock-Cooper-Munro rule for spatiotemporal learning via triplet-STDP in memristive devices. Nat. Commun..

[12] Shan X (2020). Silent synapse activation by plasma-induced oxygen vacancies in TiO_2_ nanowire-based memristor. Adv. Electron. Mater..

[13] Ke S (2021). Indium-gallium-zinc-oxide based photoelectric neuromorphic transistors for modulable photoexcited corneal nociceptor emulation. Adv. Electron. Mater..

[14] Feng G (2021). Flexible vertical photogating transistor network with an ultrashort channel for in-sensor visual nociceptor. Adv. Funct. Mater..

[15] Yao P (2020). Fully hardware-implemented memristor convolutional neural network. Nature.

[16] Roy K, Jaiswal A, Panda P (2019). Towards spike-based machine intelligence with neuromorphic computing. Nature.

[17] Pei J (2019). Towards artificial general intelligence with hybrid Tianjic chip architecture. Nature.

[18] Yin L (2020). Optically stimulated synaptic devices based on the hybrid structure of silicon nanomembrane and perovskite. Nano Lett..

[19] Yin L (2019). Synaptic silicon-nanocrystal phototransistors for neuromorphic computing. Nano Energy.

[20] Li Y (2021). Silicon-based inorganic-organic hybrid optoelectronic synaptic devices simulating cross-modal learning. Sci. China Inf. Sci..

[21] Chen C (2021). Flexible dual-gate MoS neuromorphic transistors on freestanding proton-conducting chitosan membranes. IEEE Trans. Electron Devices.

[22] He HK (2018). Photonic potentiation and electric habituation in ultrathin memristive synapses based on monolayer MoS_2_. Small.

[23] Jiang J (2019). 2D electric-double-layer phototransistor for photoelectronic and spatiotemporal hybrid neuromorphic integration. Nanoscale.

[24] John RA (2018). Synergistic gating of electro-iono-photoactive 2D chalcogenide neuristors: coexistence of hebbian and homeostatic synaptic metaplasticity. Adv. Mater..

[25] Xie S (2016). A high-quality round-shaped monolayer MoS_2_ domain and its transformation. Nanoscale.

[26] Liu, L. et al. Macroscopic-assembled-graphene nanofilms/germanium broadband photodetectors. In: *IEEE International Electron Devices Meeting (IEDM)* 194–197 (IEEE, 2021).

[27] Guo, N. et al. Light-driven WSe2-ZnO junction field-effect transistors for high-performance photodetection. *Adv. Sci*. **7**, 1901637 (2020). 10.1002/advs.201901637 PMC6947501 31921556

[28] Wang, H. et al. Memristive devices based on 2D-BiOI nanosheets and their applications to neuromorphic computing. *Appl. Phys. Lett*. **116**, 093501 (2020).

[29] Kim M (2018). Zero-static power radio-frequency switches based on MoS_2_ atomristors. Nat. Commun..

[30] Pereira RN, Rowe DJ, Anthony RJ, Kortshagen U (2011). Oxidation of freestanding silicon nanocrystals probed with electron spin resonance of interfacial dangling bonds. Phys. Rev. B—Condens. Matter Mater. Phys..

[31] Nakano H, Ishii M, Nakamura H (2005). Preparation and structure of novel siloxene nanosheets. Chem. Commun..

[32] Ohshita J (2016). Preparation and photocurrent generation of silicon nanosheets with aromatic substituents on the surface. J. Phys. Chem. C.

[33] Nakano H (2019). Silicanes modified by conjugated substituents for optoelectronic devices. Adv. Opt. Mater..

[34] Qian C (2014). Non-wettable, oxidation-stable, brightly luminescent, perfluorodecyl-capped silicon nanocrystal film. J. Am. Chem. Soc..

[35] Wang Y, Slassi A, Cornil J, Beljonne D, Samorì P (2019). Tuning the optical and electrical properties of few-layer black phosphorus via physisorption of small solvent molecules. Small.

[36] Wang Y (2019). Doping of monolayer transition-metal dichalcogenides via physisorption of aromatic solvent molecules. J. Phys. Chem. Lett..

[37] Nakano H, Nakano M, Nakanishi K, Tanaka D, Sugiyama Y (2012). Preparation of alkyl-modified silicon nanosheets by hydrosilylation. J. Am. Chem. Soc..

[38] Liu J, Yang Y, Lyu P, Nachtigall P, Xu Y (2018). Few-layer silicene nanosheets with superior lithium-storage properties. Adv. Mater..

[39] Liu X (2016). Optimum quantum yield of the light emission from 2 to 10 nm hydrosilylated silicon quantum dots. Part. Part. Syst. Charact..

[40] Tao L (2015). Silicene field-effect transistors operating at room temperature. Nat. Nanotechnol..

[41] Hirohata T, Suzuki T, Nakajima K, Mizushima Y (1993). Low-field breakdown and negative differential resistance in semi-insulating gaas. Jpn. J. Appl. Phys..

[42] Byrne JH, Hawkins RD (2015). Nonassociative learning in invertebrates. Cold Spring Harb. Perspect. Biol..

[43] Kuzum, D., Yu, S. & Philip Wong, H. S. Synaptic electronics: Materials, devices and applications. *Nanotechnology* **24**, 382001 (2013). 10.1088/0957-4484/24/38/382001 23999572

[44] Truong SN, Van Pham K, Yang W, Min KS (2016). Sequential memristor crossbar for neuromorphic pattern recognition. IEEE Trans. Nanotechnol..

[45] Diehl PU, Cook M (2015). Unsupervised learning of digit recognition using spike-timing-dependent plasticity. Front. Comput. Neurosci..

[46] Dou Z (2021). Wearable contact lens sensor for non-invasive continuous monitoring of intraocular pressure. Micromachines.

[47] Zhu J (2021). Machine learning-enabled textile-based graphene gas sensing with energy harvesting-assisted IoT application. Nano Energy.

[48] Rullen RVan, Thorpe SJ (2001). Rate coding versus temporal order coding: what the retinal ganglion cells tell the visual cortex. Neural Comput..

[49] Altavilla, C. *Methods in Neuronal Modeling: from Ions to Networks* 217–236 (MIT Press, 1999).

