# Supplementary material for: Neuromorphic device based on silicon nanosheets
Source: Nat Commun. 2022 Sep 5;13:5216. doi: 10.1038/s41467-022-32884-y (PMC9445003; doi:10.1038/s41467-022-32884-y)
Supplement: Supplementary file 1 — Supplementary Information [file 41467_2022_32884_MOESM1_ESM.pdf]

Neuromorphic Device Based on Silicon Nanosheets

Chenhao Wang, [1] Xinyi Xu, [2, 3, 4, 5] Xiaodong Pi,[1, 2] Mark D. Butala, [5] Wen Huang, [6] Lei Yin, [1] Wenbing Peng, [1] Munir Ali, [2, 3] Srikrishna Chanakya Bodepudi, [2, 3] Xvsheng Qiao, [1] Yang Xu [2, 3, 4, 5, ✉], Wei Sun, [1, ✉] Deren Yang [1, 2, ✉]

[1] State Key Laboratory of Silicon Materials & School of Materials Science and Engineering, Zhejiang University, Hangzhou 310027, PR China

[2] ZJU-Hangzhou Global Scientific and Technological Innovation Centre, Hangzhou 310027, PR China

[3] State Key Laboratory of Silicon Materials & School of Micro-Nanoelectronics, Zhejiang University, Hangzhou 310027, PR China

[4]College of Information Science and Electronics Engineering, Zhejiang University, Hangzhou 310027, PR China

[5] ZJU-UIUC Institute (ZJUI), Zhejiang University, Jiaxing 314400, PR China

[6]New Energy Technology Engineering Laboratory of Jiangsu Provence & School of Science, Nanjing University of Posts and Telecommunications, Nanjing 210023, PR China

These authors contributed equally: Chenhao Wang and Xinyi Xu

Corresponding authors: yangxu-isee@zju.edu.cn (Yang Xu),

sunnyway423@zju.edu.cn (Wei Sun),

mseyang@zju.edu.cn (Deren Yang)

Table S1 The parameter fitting result of the transient PL decay curve

Equation	$\frac{I}{I_0} = A_1 \exp\left(-\frac{t}{t_1}\right) + A_1 \exp\left(-\frac{t}{t_2}\right) + y_0$
y_0	0.02867 ± 0.00114
A_1	0.97799 ± 0.00572
t_1 [ns]	0.7563 ± 0.00778
A_2	0.03051 ± 0.00522
t_2 [ns]	4.98088 ± 1.19638
Reduced Chi-Sqr	4.05E-04
R-Square (COD)	0.97902
Adj. R-Square	0.979

Table S2 The Hall measurement results of p-fluoroaniline (pFA) modified SiNSs.

Field [G]	Resistivity [ohm-cm]	Hall Coefficient [cm^3/C]	Type	Carrier Density [1/cm^3]	Hall Mobility [cm^2/(VS)]
1.00E+03	5.69E+00	-2.73E+03	*n*	2.29E+15	3.83E+02
3.00E+03	6.39E+00	-2.43E+03	*n*	2.57E+15	3.41E+02
5.00E+03	5.83E+00	-2.38E+03	*n*	2.62E+15	3.34E+02

Table S3 The parameter fitting result of the quasi-static I-V scanning

Equation	$i_{\mathrm{ds}}(v_{\mathrm{ds}}) = \frac{V_0 C_{\mathrm{NSs}} R_{\mathrm{NSs}} R_{\mathrm{con}}}{t_0 (R_{\mathrm{NSs}} + R_{\mathrm{con}})^2}\left(1 - e^{-\frac{R_{\mathrm{NSs}}+R_{\mathrm{con}}}{C_{\mathrm{NSs}} R_{\mathrm{con}}^2}\frac{t_0}{V_0} v_{\mathrm{ds}}}\right) + \frac{v_{\mathrm{ds}}}{R_{\mathrm{NSs}} + R_{\mathrm{con}}} + i_0$
Plot	Upper Curve
C_{NSs} [F]	6.79515E-7 ± 5.89099E-22
R_{NSs} [Ω]	1.23328E9 ± 8.03124E-7
R_{con} [Ω]	6.38009E7 ± 1.54628E-8
i_0 [A]	1.70491E-9 ± 4.7859E-26
Reduced Chi-Sqr	9.35E-49
R-Square (COD)	1.00
Adj. R-Square	1.00

Equation	$i_{\mathrm{ds}}(v_{\mathrm{ds}}) = \frac{V_0 C_{\mathrm{NSs}} R_{\mathrm{NSs}} R_{\mathrm{con}}}{t_0 (R_{\mathrm{NSs}} + R_{\mathrm{con}})^2}\left(e^{-\frac{R_{\mathrm{NSs}}+R_{\mathrm{con}}}{C_{\mathrm{NSs}} R_{\mathrm{con}}^2}\frac{t_0}{V_0} v_{\mathrm{ds}}} - 1\right) + \frac{v_{\mathrm{ds}}}{R_{\mathrm{NSs}} + R_{\mathrm{con}}} + i_0'$
Plot	Lower Curve
C_{NSs} [F]	8.4414E-7 ± 6.90542E-8
R_{NSs} [Ω]	1.45625E9 ± 8.91957E7
R_{con} [Ω]	6.09062E7 ± 1.39732E6
i_0' [A]	-2.06063E-9 ± 5.20215E-12
Reduced Chi-Sqr	2.10E-21
R-Square (COD)	0.99994
Adj. R-Square	0.99994

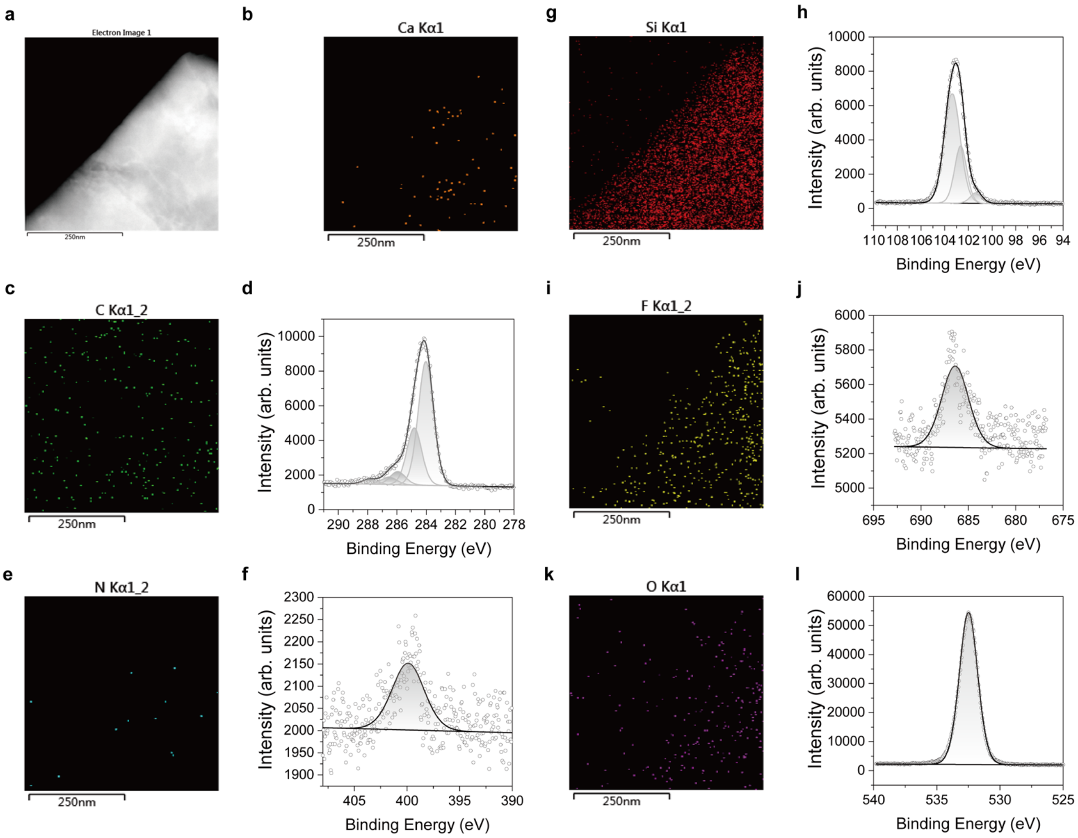

Fig. S1 Elemental analyses. **a,** A selected region for EDS. **b,** EDS mapping showing only a trace amount of residue Ca within the region. EDS mapping and XPS spectra of the modified Si-NSs for different elements: **c, d,** C, **e, f,** N, **g, h,** Si, **i, j,** F, and **k, l,** O. From EDS and XPS, F, N, C elements were detected.

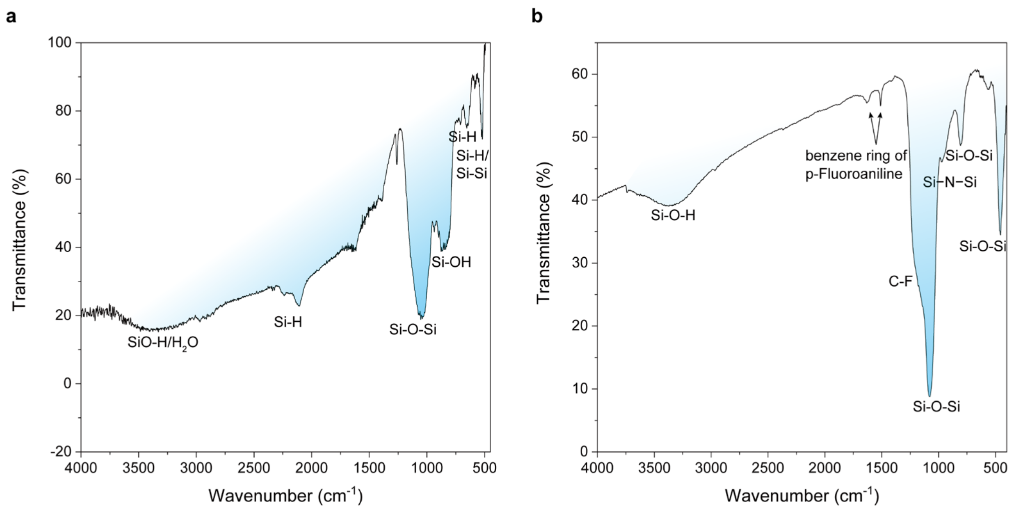

Fig. S2 FTIR spectra of Si-NSs. a, Before and **b,** after the modification of pFA, the sample was treated under 200°C in an N_2-filled glove box. Peaks for benzene ring and C-F were found after modification, which confirmed the reaction with pFA.

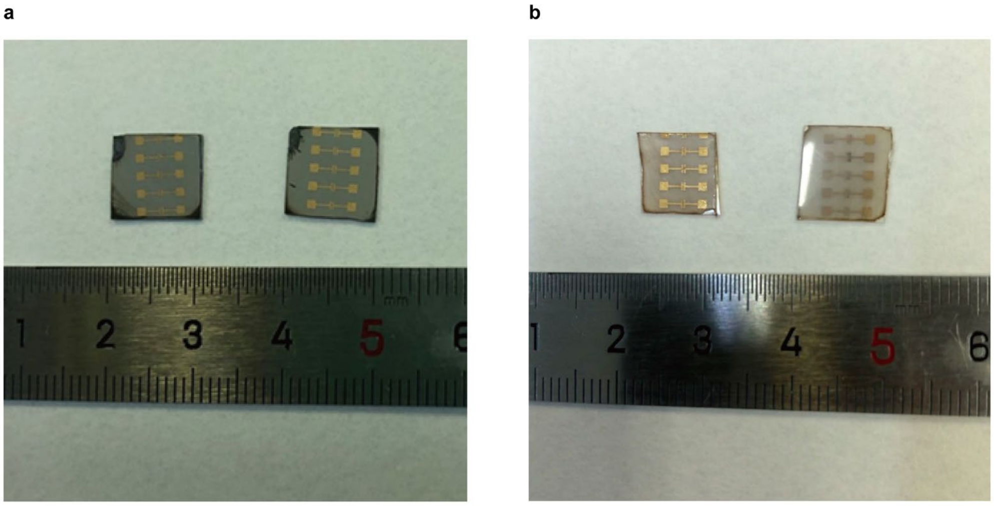

Fig. S3 Digital photographs of devices. a, Devices fabricated on Si substrate and **b,** PET. The one on the right is placed with the backside upwards.

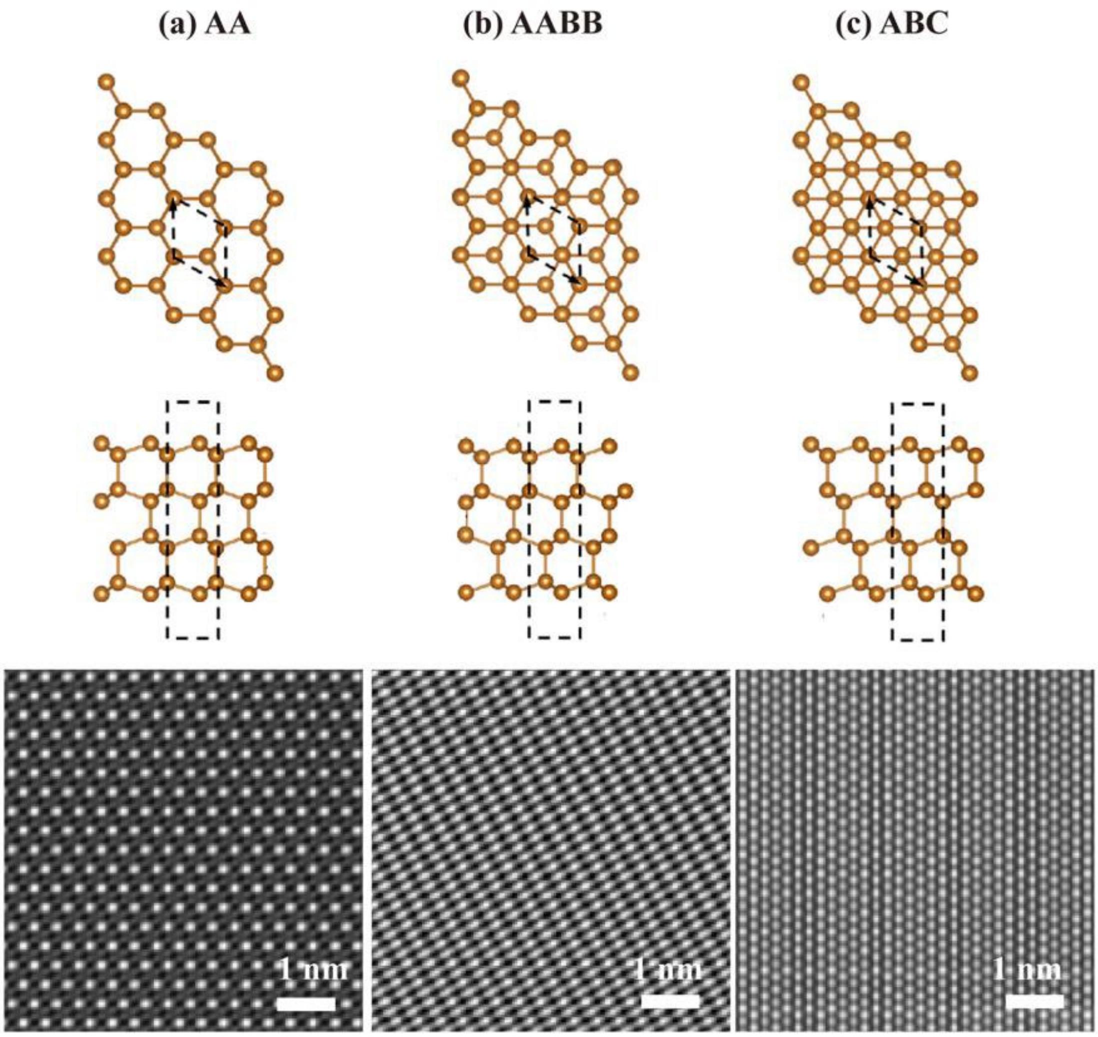

Fig. S4 Top (upper panel) and side views (lower panel) of the geometric structures of four layer silicene with three common stacking modes and their respective calculated HRTEM images. **a,** Eclipsed AA, **b,** staggered AABB, and **c,** staggered ABC. (Reproduced with permission. Copyright 2018, Wiley-VCH Verlag GmbH & Co. KGaA, Weinheim.[1])

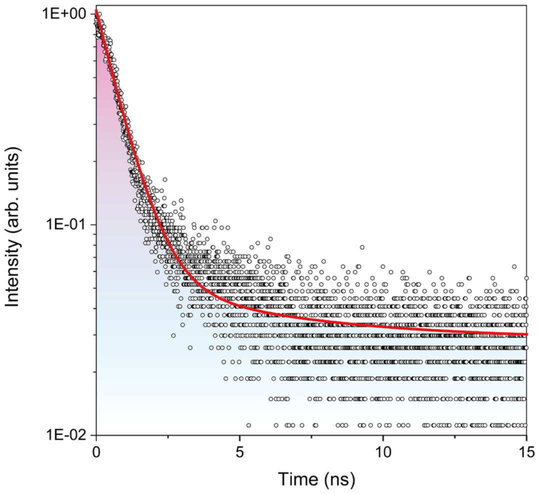

Fig. S5 Transient PL decay curve of Si-NSs. A 280 nm laser was used to excite the Si-NSs and the monitoring wavelength was 350 nm. The dots were the experimental result and the red line was the parameter fitting result. The lifetime calculated from this curve is given in Table S1.

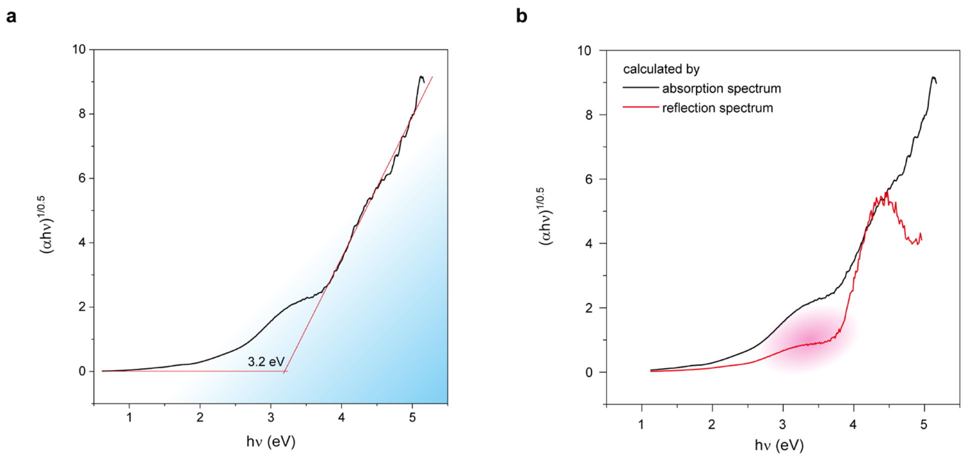

Fig. S6 The Tauc plot for bandgap calculation. a, The UV-vis absorption spectrum was used to calculate the direct bandgap of Si-NSs. **b,** The comparison of Tauc plots obtained from two different modes. The red line was calculated by reflection spectrum and the black was by absorption spectrum.

Due to the chemical exfoliation method for the preparation of SiNSs, the presence of some impurity in the active material is inevitable, which might result in the small shoulder in the Tauc plot, often seen for many semiconductor nanomaterials chemically synthesized. Moreover, we think the diffuse reflection of the film also contributed to the shoulder. As the film was not mirror-like smooth, the diffuse reflection led to the discrepancy, when we used the absorption spectrum for calculation. Tauc plot calculated by refection spectrum counted some of the reflected photons. The Tauc plot calculated by reflection spectra possessed a much less significant shoulder effect, compared to the absorption spectrum (Figure S6(b)). Nevertheless, due to the ineluctable scattering and intragap states, the shoulder cannot be fully eliminated.

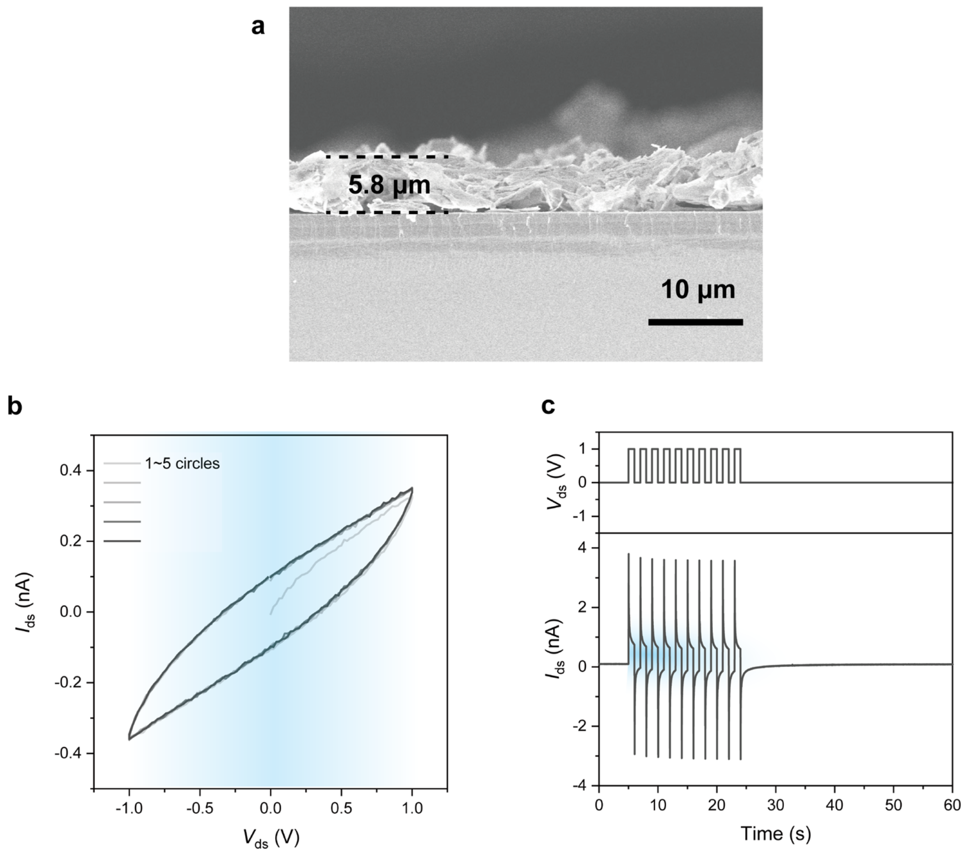

Fig. S7 The performance of the device with a thinner channel. a, The cross-section SEM of the device with the thickness of 5.8 μm. **b,** The quasi-static I-V scanning and **c,** the synaptic behavior of the device.

The 15 μm thick channels of partial oxidized hierarchical stacking SiNSs bundles could be regarded as a large number of parallel capacitors, which contributed to large gross capacitance and the carrier storage ability. Subsequently, we took advantage of the capacitance to fabricate the synaptic device. The channel thickness of 15 μm was chosen within an optimal range and with consideration of the vertical potential distribution in an equivalent circuit, for a proper match of the capacitance and resistance. Especially, the larger capacitance contributed to the strong charge storage ability and good synaptic behavior.

We fabricated devices with different channel thicknesses, to investigate the impact of channel thickness. The device with a thinner channel (5.8 μm, Figure S7 (a)) had a similar I-V hysteresis loop and synaptic response, Figure S7 (b-c). However, the weight change was less significant compared to the devices with a thicker channel mentioned in the manuscript. Also, the large resistance led to the revealing of contact capacitance between the probe and devices, resulting in large discharge spikes at the rising edge and larger measurement errors. The thicker channel contributes to a larger capacitance and smaller resistance according to Equation S1~2 (simple linear resistance and parallel capacitor model were applied for estimation). The capacitance is proportional

to the device thickness, and resistance is inversely proportional to thickness. Therefore, a larger thickness was preferred.

$$C = \frac{d_{\text{device}}}{d_{\text{bundle}}} \frac{\varepsilon_0 \varepsilon_r S_{\text{device}}}{d_{\text{bundle}}} \quad \text{(S1)}$$

$$R = \rho \frac{l_{\text{device}}}{d_{\text{device}} b_{\text{device}}} \quad \text{(S2)}$$

While too thick channel took a large amount of SiNSs and might be unfeasible for large-scale device fabrication. Also, we considered devices' vertical electric field distribution like the fringe effect of electrode and nonideality of the parallel capacitance model. The vertical field effect is more significant in thicker devices.

For the present lateral device layout, the curvature and vertical distribution of the electric field were unavoidable for the present lateral layout. Therefore, the discrepancy between the extracted capacitance parameters from the model (7×10^{-7} F), and the calculated one by device dimension (1×10^{-7} F) could result from the vertical electric field distribution. The simple models (Equation S1~2) were more like the qualitative analysis. And the actual device fabrication and performance measurement were necessary.

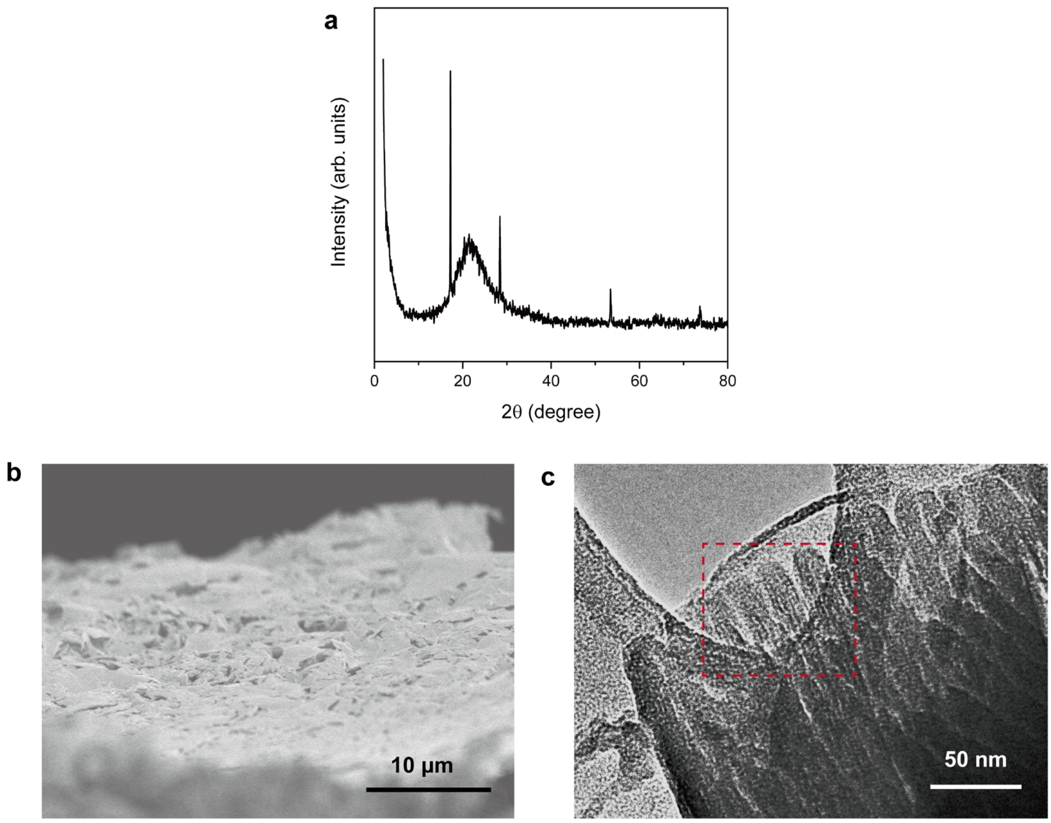

Fig. S8 Characterization of SiNSs after thermal treatment under 200 °C. a, The XRD of SiNSs treated under 200 °C. **b,** SEM of the SiNSs active layer of device treated under 200 °C. **c,** TEM of the stacking structure. The hierarchical structure demonstrates the SiNS first formed bundles and further formed higher-level structures.

The attachment of organic modifier and hierarchical structure of SiNSs retained after the thermal treatment at 200 °C. We provided the XRD and SEM results of SiNSs (Figure S8(a-b)). After 200 °C treatment, the four XRD peaks were retained, which indicated the SiNSs structure did not change after thermal treatment (Figure S8(a)). The SEM demonstrated a device cross-section structure of active SiNSs layers (Figure S8(b)). The TEM of SiNSs treated under 200 °C (Figure S8(c)) revealed the stacking of lamellar layers of SiNSs, which confirmed the hierarchical structure proposed in the manuscript. Few layers first stick to each other and form bundles, while there were many interval gaps between bundles. The hierarchical structure broke the long-range order of stack, which resulted in no peak observed in XRD attributed to the stacking structure.

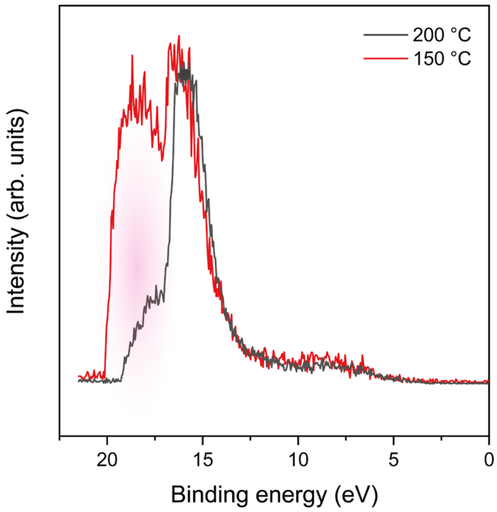

Fig. S9 The comparison of UPS for samples treated under different temperatures. The black line is for the sample treated under 200 °C as demonstrated in the manuscript. Whereas, the red line corresponds to the sample treated under 150 °C which exhibited a higher oxidation level.

The poor arrangement of SiNSs and the remaining base solvent (amine) could stimulate oxidation. Therefore, the higher thermal treatment temperature above the boiling point (187 °C) of the solvent (pFA) in a N_2-filled glove box improved the arrangement of SiNSs and eliminated the solvent. Although the oxidation of SiNSs could not be fully prevented, it was ameliorated when increasing the thermal treatment temperature. The thermal-treatment-induced elimination of oxidation was proved by UPS. The channels of the devices treated at 150 °C and 200 °C were characterized by UPS. We found that both of the devices had a shoulder for partial oxidation. Compared with the 150 °C, the device treated at 200 °C has less significant shoulder, which indicated the thermal treatment at a higher temperature can reduce the oxidation.

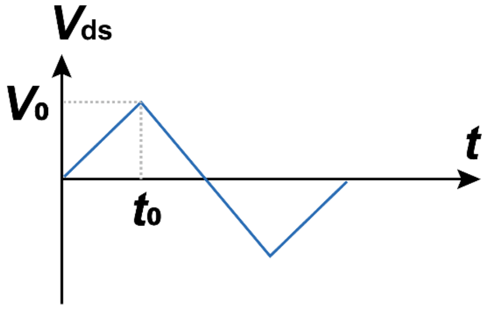

Fig. S10 Schematic figure for I-V curve analytical solution calculation. Triangular signal vs. time for quasi-static I-V scanning.

The derivation of the formula for the quasi-static curve of the device:

$$
\begin{aligned}
v_{\mathrm{ds}} &= v_{\mathrm{NSs}} + v_{\mathrm{con}} \\
&= i_{R_{\mathrm{NSs}}} R_{\mathrm{NSs}} + i_{\mathrm{ds}} R_{\mathrm{con}} \\
&\overset{i_{R_{\mathrm{NSs}}} + i_{C_{\mathrm{NSs}}} = i_{\mathrm{ds}}}{=\!=\!=\!=} i_{\mathrm{ds}}(R_{\mathrm{NSs}} + R_{\mathrm{con}}) - i_{C_{\mathrm{NSs}}} R_{\mathrm{con}} \\
&\overset{C_{\mathrm{NSs}} = \frac{q_{C_{\mathrm{NSs}}}}{v_{\mathrm{NSs}}}}{\underset{C_{\mathrm{NSs}} \frac{dv_{\mathrm{NSs}}}{dt} = i_{C_{\mathrm{NSs}}}}{=\!=\!=\!=}} i_{\mathrm{ds}}(R_{\mathrm{NSs}} + R_{\mathrm{con}}) - C_{\mathrm{NSs}} \frac{dv_{\mathrm{NSs}}}{dt} R_{\mathrm{con}} \\
&\overset{v_{\mathrm{NSs}} = v_{\mathrm{ds}} - i_{\mathrm{ds}} R_{\mathrm{con}}}{=\!=\!=\!=} i_{\mathrm{ds}}(R_{\mathrm{NSs}} + R_{\mathrm{con}}) - C_{\mathrm{NSs}} \frac{d(v_{\mathrm{ds}} - i_{\mathrm{ds}} R_{\mathrm{con}})}{dt} R_{\mathrm{con}}
\end{aligned}
$$

$$
\frac{di_{\mathrm{ds}}(t)}{dt} + \frac{R_{\mathrm{NSs}} + R_{\mathrm{con}}}{C_{\mathrm{NSs}} R_{\mathrm{con}}{}^2} i_{\mathrm{ds}}(\mathrm{t}) = \frac{v_{\mathrm{ds}}(t)}{C_{\mathrm{NSs}} R_{\mathrm{con}}{}^2} + \frac{1}{R_{\mathrm{con}}} \frac{dv_{\mathrm{ds}}(t)}{dt}
$$

$(0, t_0)$

$$
v_{\mathrm{ds}} = \frac{V_0}{t_0} t; \ \frac{dv_{\mathrm{ds}}}{dt} = \frac{V_0}{t_0}
$$

$$
\frac{di_{\mathrm{ds}}(t)}{dt} + \frac{R_{\mathrm{NSs}} + R_{\mathrm{con}}}{C_{\mathrm{NSs}} R_{\mathrm{con}}{}^2} i_{\mathrm{ds}}(t) = \frac{V_0}{t_0 C_{\mathrm{NSs}} R_{\mathrm{con}}{}^2} t + \frac{V_0}{t_0 R_{\mathrm{con}}}
$$

$$
i_{\mathrm{ds}}(t) = \tilde{c} e^{-\frac{R_{\mathrm{NSs}} + R_{\mathrm{con}}}{C_{\mathrm{NSs}} R_{\mathrm{con}}{}^2} t} + \frac{V_0}{t_0(R_{\mathrm{NSs}} + R_{\mathrm{con}})} t + \frac{\mathrm{V}_0 C_{\mathrm{NSs}} R_{\mathrm{NSs}} R_{\mathrm{con}}}{t_0(R_{\mathrm{NSs}} + R_{\mathrm{con}})^2}
$$

Boundary condition: $i(0) = 0$

$$
i_{\mathrm{ds}}(t) = \frac{\mathrm{V}_0 C_{\mathrm{NSs}} R_{\mathrm{NSs}} R_{\mathrm{con}}}{t_0(R_{\mathrm{NSs}} + R_{\mathrm{con}})^2} \left(1 - e^{-\frac{R_{\mathrm{NSs}} + R_{\mathrm{con}}}{C_{\mathrm{NSs}} R_{\mathrm{con}}{}^2} t}\right) + \frac{V_0}{t_0(R_{\mathrm{NSs}} + R_{\mathrm{con}})} t
$$

$$
i_{\mathrm{ds}}(v_{\mathrm{ds}}) = \frac{\mathrm{V}_0 C_{\mathrm{NSs}} R_{\mathrm{NSs}} R_{\mathrm{con}}}{t_0(R_{\mathrm{NSs}} + R_{\mathrm{con}})^2} \left(1 - e^{-\frac{R_{\mathrm{NSs}} + R_{\mathrm{con}}}{C_{\mathrm{NSs}} R_{\mathrm{con}}{}^2} \frac{t_0}{V_0} v_{\mathrm{ds}}}\right) + \frac{v_{\mathrm{ds}}}{R_{\mathrm{NSs}} + R_{\mathrm{con}}}
$$

$((4n+1)t_0, (4n+3)t_0) \ \mathrm{n} \in \mathrm{N}, \mathrm{n} \to +\infty$

Let: $t^* = t - (4n+2)t_0$

$$v_{\text{ds}} = -\frac{V_0}{t_0}t^*;\ \frac{dv}{dt} = -\frac{V_0}{t_0}$$

$$\frac{di_{\text{ds}}(t^*)}{dt} + \frac{R_{\text{NSs}} + R_{\text{con}}}{C_{\text{NSs}}R_{\text{con}}{}^2} i_{\text{ds}}(t^*) = -\frac{V_0}{t_0 C_{\text{NSs}}R_{\text{con}}{}^2}t - \frac{V_0}{t_0 R_{\text{con}}}$$

$$i_{\text{ds}}(t^*) = \tilde{c}e^{-\frac{R_{\text{NSs}}+R_{\text{con}}}{C_{\text{NSs}}R_{\text{con}}{}^2}t^*} - \frac{V_0}{t_0(R_{\text{NSs}} + R_{\text{con}})}t^* - \frac{V_0 C_{\text{NSs}}R_{\text{NSs}}R_{\text{con}}}{t_0(R_{\text{NSs}} + R_{\text{con}})^2}$$

Boundary condition: $\lim_{t^*\to -t_0} i(t^*) = \lim_{t^*\to t_0} -i(t^*)$

because the hysteresis curve is closed and exhibits central symmetry.

$$\tilde{c}\left(e^{-\frac{R_{\text{NSs}}+R_{\text{con}}}{C_{\text{NSs}}R_{\text{con}}{}^2}t_0} + e^{\frac{R_{\text{NSs}}+R_{\text{con}}}{C_{\text{NSs}}R_{\text{con}}{}^2}t_0}\right) = 2\frac{V_0 C_{\text{NSs}}R_{\text{NSs}}R_{\text{con}}}{t_0(R_{\text{NSs}} + R_{\text{con}})^2}$$

Since $t_0 = 4$s, and is much less than the characteristic lifetime of the device, i.e., $\frac{R_{\text{NSs}}+R_{\text{con}}}{C_{\text{NSs}}R_{\text{con}}{}^2}$ and C_{NSs} are large for the SiNSs, so $e^{-\frac{R_{\text{NSs}}+R_{\text{con}}}{C_{\text{NSs}}R_{\text{con}}{}^2}t_0} + e^{\frac{R_{\text{NSs}}+R_{\text{con}}}{C_{\text{NSs}}R_{\text{con}}{}^2}t_0} \approx 2, \tilde{c} \approx \frac{V_0 C_{\text{NSs}}R_{\text{NSs}}R_{\text{con}}}{t_0(R_{\text{NSs}}+R_{\text{con}})^2}$

In addition, we assume there is a small deviation term i_0, when $t^* = 0$.

$$i_{\text{ds}}(t^*) = \frac{V_0 C_{\text{NSs}}R_{\text{NSs}}R_{\text{con}}}{t_0(R_{\text{NSs}} + R_{\text{con}})^2}\left(e^{-\frac{R_{\text{NSs}}+R_{\text{con}}}{C_{\text{NSs}}R_{\text{con}}{}^2}t^*} - 1\right) + \frac{V_0}{t_0(R_{\text{NSs}} + R_{\text{con}})}t^* + i_0$$

$$i_{\text{ds}}(v_{\text{ds}}) = \frac{V_0 C_{\text{NSs}}R_{\text{NSs}}R_{\text{con}}}{t_0(R_{\text{NSs}} + R_{\text{con}})^2}\left(e^{-\frac{R_{\text{NSs}}+R_{\text{con}}}{C_{\text{NSs}}R_{\text{con}}{}^2}\frac{t_0}{V_0}v_{\text{ds}}} - 1\right) + \frac{v_{\text{ds}}}{R_{\text{NSs}} + R_{\text{con}}} + i_0$$

Similarly, for $((4n+3)t_0, (4n+5)t_0)$ $n \in N, n \to +\infty$

Let: $t^* = t - (4n+4)t_0$

$$i_{\text{ds}}(t^*) = \frac{V_0 C_{\text{NSs}}R_{\text{NSs}}R_{\text{con}}}{t_0(R_{\text{NSs}} + R_{\text{con}})^2}\left(1 - e^{-\frac{R_{\text{NSs}}+R_{\text{con}}}{C_{\text{NSs}}R_{\text{con}}{}^2}t}\right) - \frac{V_0}{t_0(R_{\text{NSs}} + R_{\text{con}})}t^* + i_0'$$

$$i_{\text{ds}}(v_{\text{ds}}) = \frac{V_0 C_{\text{NSs}}R_{\text{NSs}}R_{\text{con}}}{t_0(R_{\text{NSs}} + R_{\text{con}})^2}\left(1 - e^{\frac{R_{\text{NSs}}+R_{\text{con}}}{C_{\text{NSs}}R_{\text{con}}{}^2}\frac{t_0}{V_0}v_{\text{ds}}}\right) + \frac{v_{\text{ds}}}{R_{\text{NSs}} + R_{\text{con}}} + i_0'$$

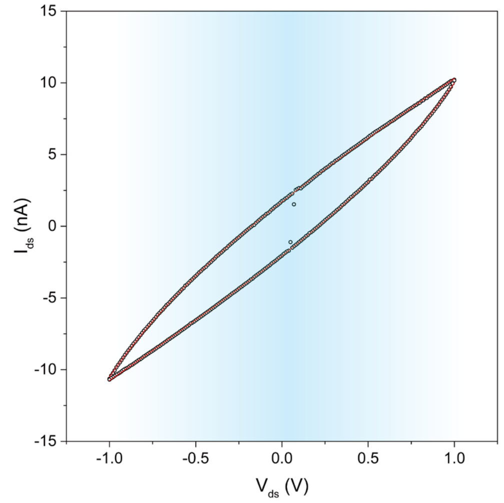

Fig. S11 Fitting of quasi-static I-V scanning based on the proposed model. The dots were the experimental result and the red line is the fitting result. The fitting parameters are given in Table S3.

We estimated the capacitance according to the device dimension. Notably, according to parameter fitting, the device capacitance was fairly large within a small channel (100 μm * 1000 μm). Whereas, using the parallel plate capacitor model (equation S1), the capacitance was ~0.2 pF, which is distinctively smaller than the capacitance from the fitting.

$$C = \frac{\varepsilon_0 \varepsilon_r S}{d} \quad \text{(S1)}$$

where ε_0 is the vacuum permittivity, ε_r is the relative permittivity of the active layer, *S* is the area of the parallel plane, and *d* is the thickness.

We attributed the large capacitance to connected capacitors of layered partially oxidized SiNSs bundles, which were both vertically and horizontally attached to each other in the channel. The outer shells of bundles converted into SiO_2 due to the partial oxidation, which contributed to the capacitance. While the inner cores remained intact, which contributed to the conduction. Thus, considering the dimension of the channel and the SiNSs to estimate the number of bundles (n_{bundle}) , the device capacitance was better estimated as ~1 * 10^{-7} F (equation S2), which was close to the previously extrapolated result from fitting.

$$C = n_{\mathrm{bundle}} \frac{\varepsilon_0 \varepsilon_{\mathrm{r}} S_{\mathrm{bundle}}}{d_{\mathrm{bundle}}} = n_{\mathrm{vertical}} n_{\mathrm{lateral}} \frac{\varepsilon_0 \varepsilon_{\mathrm{r}} S_{\mathrm{bundle}}}{d_{\mathrm{bundle}}} = \frac{d_{\mathrm{device}} S_{\mathrm{device}}}{d_{\mathrm{bundle}} S_{\mathrm{bundle}}} \frac{\varepsilon_0 \varepsilon_{\mathrm{r}} S_{\mathrm{bundle}}}{d_{\mathrm{bundle}}} = \frac{d_{\mathrm{device}}}{d_{\mathrm{bundle}}} \frac{\varepsilon_0 \varepsilon_{\mathrm{r}} S_{\mathrm{device}}}{d_{\mathrm{bundle}}}$$

(S2)

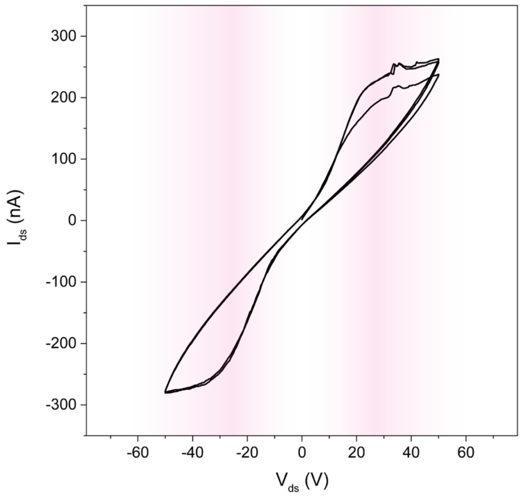

Fig. S12 Quasi-static I-V curves of the fabricated devices on the PET substrate. The devices demonstrated a similar memristor-like behavior as the Si substrate ones in the manuscript.

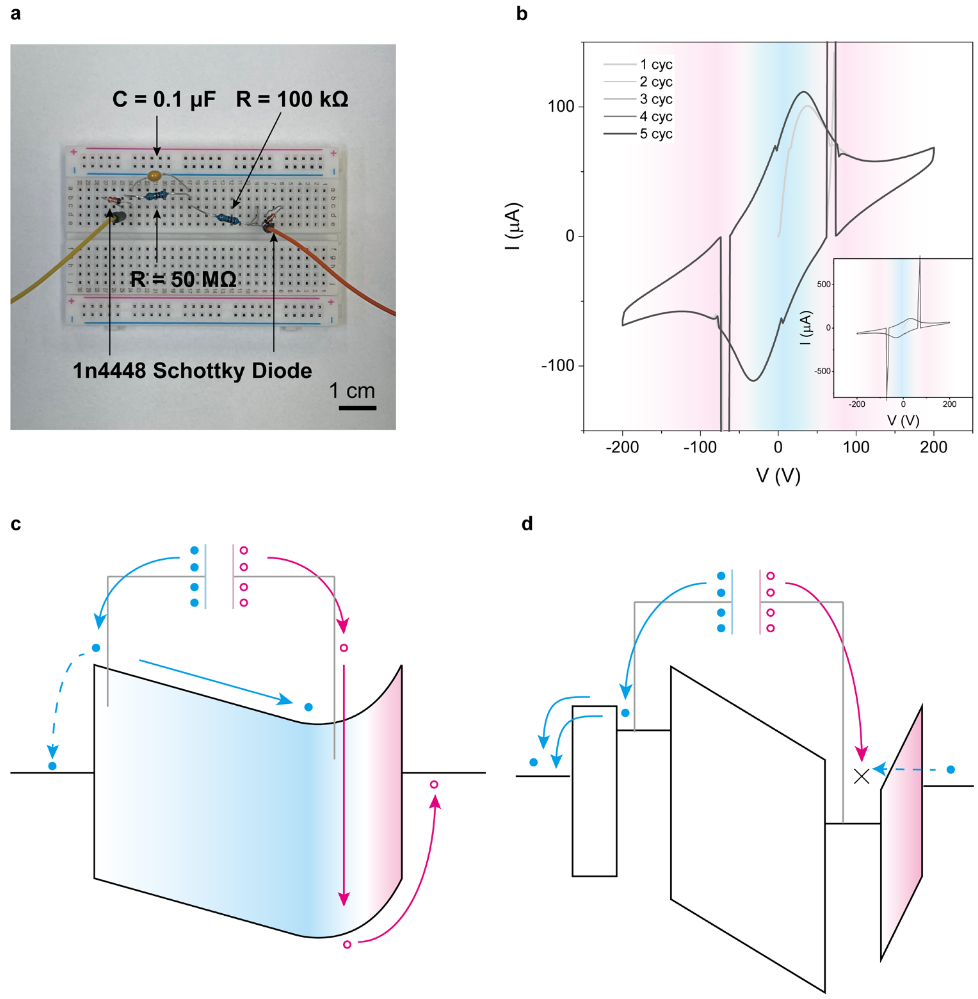

Fig. S13 Replication of memristor behavior by electronic components. a, Digital picture of the electronic components mimicking the memristor behavior of the SiNS devices we fabricated. **b,** Quasi-static I-V curves of the electronic components which replicated the memristor behavior. Schematic figures demonstrate the difference between **c,** the devices and **d,** the electronic components. The blue dots correspond to the electrons, while red circles correspond to the holes. The blue region of the band diagram corresponds to the negative charge-rich region due to the accumulation of electrons, while the red region corresponds to the depletion region.

We observed a sudden large leakage current for the commercial electronic components when the current was reduced to 0, which is different from the device we fabricated. This phenomenon was due to the fact that discrete diode components change to the flat band mode. Then, the electron stored in the capacitor could easily deplete through the diode (Figure S10 (d)). Whereas, for the Si-NSs device, the Schottky junction could never be flat band mode. The Schottky diode was formed by SiNSs, and some carriers could be stored at the junction which was attributed to the tilt of the band. Thus, the carriers in the SiNSs could not get back to the electrode (Figure S10 (c)).

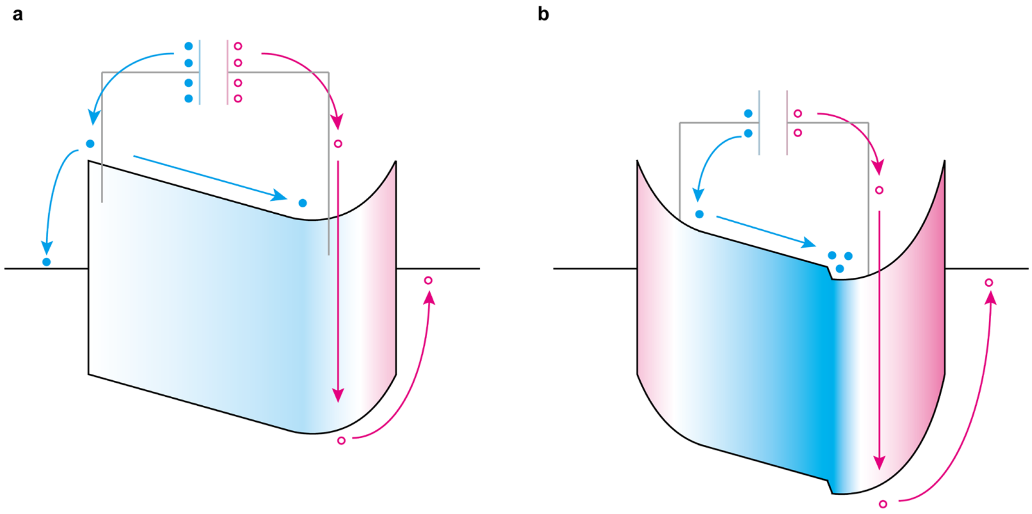

Fig. S14 Schematic figure showing the shift of charges. a, Before and **b,** after the interval between measurements.

The first cycle was different from subsequent cycles in that the threshold voltage for switching from LRS to HRS of the first cycle was smaller. This was due to the fact that during the measurement interval (Figure 2(h)), the charge stored in the C_{NSs} from the former measurement transferred to the Schottky junction. Due to the stored carriers in the C_{NSs}, the voltage (v_{NSs}) across the SiNSs stacks (C_{NSs} and R_{NSs}) would not suddenly disappear. During the measurement interval without applied voltage (v_{ds}), existing v_{NSs} caused the carrier drift and redistribution (Figure S12(a)). The holes transferred to the electrode without hindrance, while the Schottky barrier impeded the movement of electrons. Driven by the electric field, electrons accumulated at one side of the Schottky junction, which had higher electric potential. To balance the negative charges of accumulated electrons at the junction, the space charge region with positive charges enlarged. The redistribution of carrier impacted the bandgap and electric field near the junctions. The larger electric field facilitated the breakdown under a lower applied voltage (Figure S12(b)). Therefore, the NDR effect and charging of the C_{NSs} happened at a lower voltage for the first cycle. The redistribution prevented the complete varnishing of stored information in the memristor but led to the difference between the first cycle and subsequent cycles.

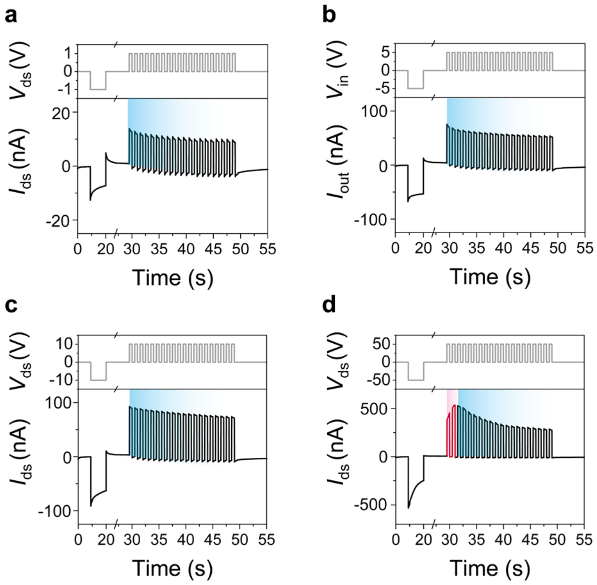

Fig. S15 Synaptic behaviors of the devices subjected to spikes of different voltages. a, 1V, **b,** 5V, **c,** 10V, **d,** 50V. The portion in red corresponds to the abnormal PPF spikes.

For synapses, the synaptic weight refers to the signal intensity transmitted from the presynaptic to the postsynaptic neuron. In nervous systems, the synaptic weight would be automatically tuned by time intervals and rates of the spikes passing through the synapses. These processes are believed highly connected to learning and memory. Our devices could mimic these fundamental functions.

For our devices, the applied voltage V_{ds} on the drain was the input signal, and the channel current I_{ds} of devices could be referred to as the synaptic weight. The current at the falling edge of the voltage spikes was measured to avoid the non-ideality at the rising edge (the capacitance between test probe and devices could lead to irregular current gain). The applied spike voltage was as low as 1 V to ensure low energy consumption of the device per spike (~10^{-8} J), which is an important merit of neuromorphic computation. Also, under low voltage, the explicit synaptic behaviors and could be observed (Figure S12 (a-c)) without large contact resistance variation. When the applied voltage was large, the NDR effect could cause the abnormal PPF for the first several spikes(Figure S12 (d)). Meanwhile, the duration and interval of the spike were all set as long as 1 s, considering the limited measurement precision.

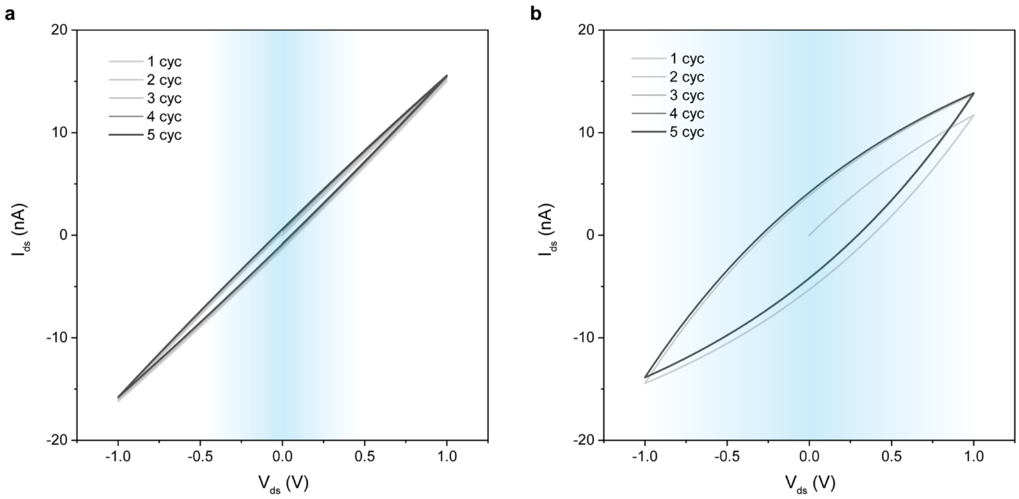

Fig. S16 Simulated quasi-static I-V curves. a, The simulation results with capacitance C_{NSs} = 7 * 10^{-7} F and resistance R_{NSs} = 1 * 10^9 Ω and R_{con} = 6 * 10^7 Ω set according to the fitting (Figure S9, Table S2). **b,** The simulation was closer to the experimental result (Figure 2(f)), with an adjusted capacitance value (C_{NSs} = 1 * 10^{-7} F).

For the simulation, we tuned the value of C_{NSs} to 1 * 10^{-7} F for a better match. We found the boundary condition could be largely varied due to the actual non-constant r_{con}, which significantly impacted the fitted value of C_{NSs} discussed in the quasi-static I-V measurement. After adjusting C_{NSs}, the simulated I-V loop also corresponded well with the experimental result (Figure 2(f)).

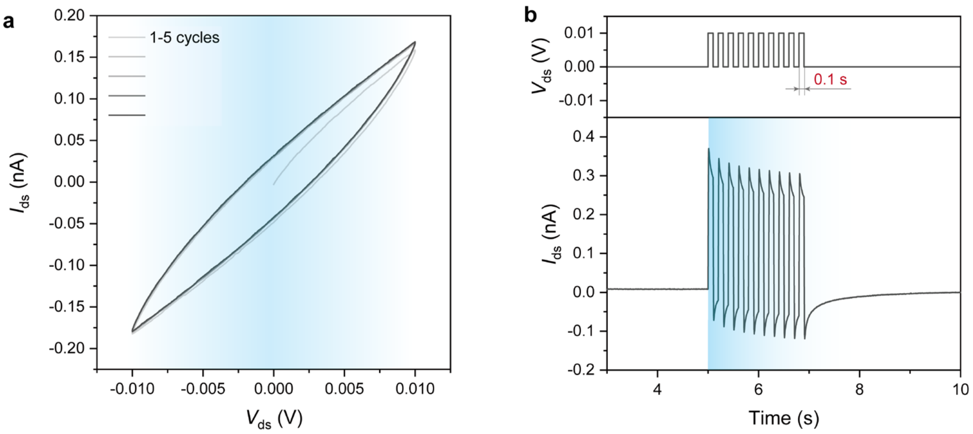

Fig. S17 The characterization of the device fabricated using SiNSs with a different modifier. **a,** The quasi-static I-V scanning of and **b,** the synaptic behavior of the device.

In the manuscript, we mainly focused on the discussion of the mechanism of the devices. The energy efficiency and function speed are surely critical parameters of neuromorphic devices. For these two parameters, our device was far behind those of the state-of-the-art devices (e.g. Zero-static power MoS_2 atomristors enable high-frequency switches that operate at 50 GHz[2]). Therefore, one of the aims of our ongoing work is to develop new and specific synthesis to modify the materials to fabricate the device with better performance. Figure R6 demonstrated the device using the modified SiNSs had similar I-V and synaptic characteristics as the devices mentioned in the manuscript. Meanwhile, it possessed much lower energy consumption (~0.3 pJ (= 0.3 s * 0.01 V * 10^{-10} A)), with a higher working frequency (5 Hz), which might potentially support SiNSs' further realistic application. Nevertheless, since the relevance of improvement of work frequency and power consumption is comparatively less significant to the focus of this work, we intend to report these details separately in future work.

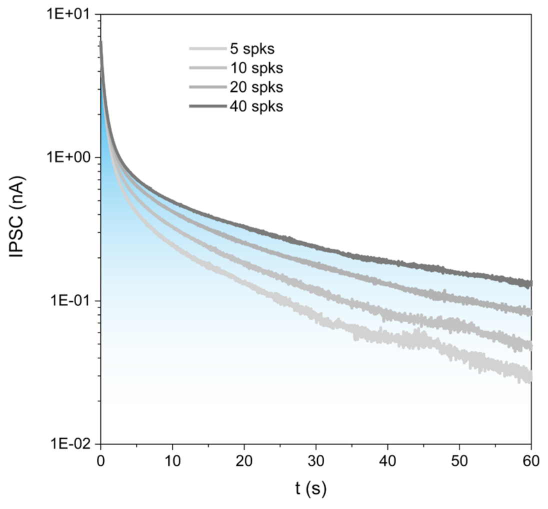

Fig. S18 The leaky current of the device after several consecutive 1 V voltage spikes. The spike rate was 0.5 Hz. The duration and the interval time were both 1s.

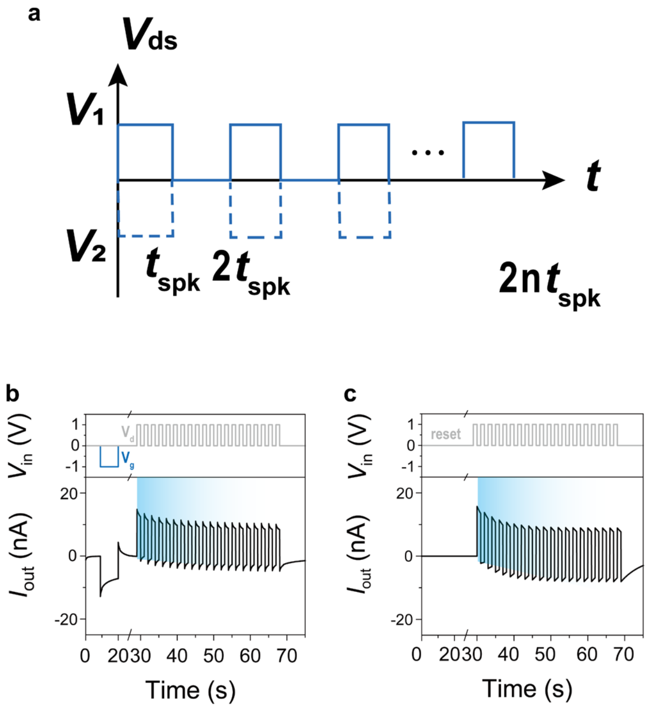

Fig. S19 Schematic figure for LTM analytical solution calculation. a, Voltage spikes applied to the device for LTM. **b,** The experimental result. **c,** The simulation result.

LTM corresponds to the ability to maintain the synaptic weight, which can be referred to as synaptic plasticity involved in learning and memory activities. The following is the derivation of the formula for the LTM:

$$\begin{aligned} v_{\mathrm{ds}} &= v_{\mathrm{NSs}} + v_{\mathrm{con}} \\ &= i_{R_{\mathrm{NSs}}} R_{\mathrm{NSs}} + i_{\mathrm{ds}} R_{\mathrm{con}} \\ &= i_{R_{\mathrm{NSs}}} R_{\mathrm{NSs}} + \left(i_{C_{\mathrm{NSs}}} + i_{R_{\mathrm{NSs}}}\right) R_{\mathrm{con}} \end{aligned}$$

$$\overset{C_{\mathrm{NSs}} = \frac{q_{C_{\mathrm{NSs}}}}{v_{\mathrm{NSs}}}}{\underset{C_{\mathrm{NSs}} \frac{dv_{\mathrm{NSs}}}{dt} = i_{C_{\mathrm{NSs}}}}{=\!=\!=\!=\!=}} \frac{v_{\mathrm{NSs}}}{R_{\mathrm{NSs}}}\left(R_{\mathrm{NSs}} + R_{\mathrm{con}}\right) + \frac{Cdv_{\mathrm{NSs}}}{dt} R_{\mathrm{con}}$$

$$\frac{dv_{\mathrm{NSs}}(t)}{dt} + \frac{R_{\mathrm{NSs}} + R_{\mathrm{con}}}{C_{\mathrm{NSs}} R_{\mathrm{NSs}} R_{\mathrm{con}}} v_{\mathrm{NSs}}(t) = v_{\mathrm{ds}}(t)$$

When $(v_{\mathrm{ds}} = V_{\mathrm{spk}})$,

$$v_{\mathrm{NSs}}(t) = \tilde{c} e^{-\frac{R_{\mathrm{NSs}} + R_{\mathrm{con}}}{C_{\mathrm{NSs}} R_{\mathrm{con}}{}^{2}} t} + \frac{R_{\mathrm{NSs}} V_{\mathrm{spk}}}{R_{\mathrm{NSs}} + R_{\mathrm{con}}}$$

When $(v_{\mathrm{ds}} = 0)$,

$$v_{\mathrm{NSs}}(t) = \tilde{c} e^{-\frac{R_{\mathrm{NSs}} + R_{\mathrm{con}}}{C_{\mathrm{NSs}} R_{\mathrm{con}}{}^{2}} t}$$

$(0, t_{\mathrm{spk}})$

$$v_{\mathrm{NSs}}(t) = \frac{R_{\mathrm{NSs}} V_{\mathrm{spk}}}{R_{\mathrm{NSs}} + R_{\mathrm{con}}} \left(1 - e^{-\frac{R_{\mathrm{NSs}} + R_{\mathrm{con}}}{C_{\mathrm{NSs}} R_{\mathrm{con}}{}^{2}} t}\right)$$

$$v_{\mathrm{NSs}}(t_{\mathrm{spk}}) = \frac{R_{\mathrm{NSs}}V_{\mathrm{spk}}}{R_{\mathrm{NSs}} + R_{\mathrm{con}}}\left(1 - e^{-\frac{R_{\mathrm{NSs}}+R_{\mathrm{con}}}{C_{\mathrm{NSs}}R_{\mathrm{con}}^{2}}t_{\mathrm{spk}}}\right)$$

Let $\frac{R_{\mathrm{NSs}}V_{\mathrm{spk}}}{R_{\mathrm{NSs}}+R_{\mathrm{con}}} = A,\ e^{-\frac{R_{\mathrm{NSs}}+R_{\mathrm{con}}}{C_{\mathrm{NSs}}R_{\mathrm{con}}^{2}}t_{\mathrm{spk}}} = B$

$$v_{\mathrm{NSs}}(t_{\mathrm{spk}}) = A(-B + 1)$$

$(t_{\mathrm{spk}}, 2t_{\mathrm{spk}})$

$$t^{*} = t - t_{\mathrm{spk}}$$

$$v_{\mathrm{NSs}}(t^{*}) = A(-B + 1)e^{-\frac{R_{\mathrm{NSs}}+R_{\mathrm{con}}}{C_{\mathrm{NSs}}R_{\mathrm{con}}^{2}}t^{*}}$$

$$v_{\mathrm{NSs}}(t^{*} = t_{\mathrm{spk}}) = A(-B + 1)e^{-\frac{R_{\mathrm{NSs}}+R_{\mathrm{con}}}{C_{\mathrm{NSs}}R_{\mathrm{con}}^{2}}t_{\mathrm{spk}}} = A(-B^{2} + B)$$

$(2t_{\mathrm{spk}}, 3t_{\mathrm{spk}})$

$$t^{*} = t - 2t_{0}$$

$$v_{\mathrm{NSs}}(t^{*}) = A(-B^{2} + B - 1)e^{-\frac{R_{\mathrm{NSs}}+R_{\mathrm{con}}}{C_{\mathrm{NSs}}R_{\mathrm{con}}^{2}}t^{*}} + A$$

$$v_{\mathrm{NSs}}(t^{*} = t_{\mathrm{spk}}) = A(-B^{3} + B^{2} - B + 1)$$

$(3t_{\mathrm{spk}}, 4t_{\mathrm{spk}})$

$$t^{*} = t - 3t_{\mathrm{spk}}$$

$$v_{\mathrm{NSs}}(t^{*} = t_{\mathrm{spk}}) = A(-B^{4} + B^{3} - B^{2} + B)$$

$(4t_{\mathrm{spk}}, 5t_{\mathrm{spk}})$

$$t^{*} = t - 4t_{\mathrm{spk}}$$

$$v_{\mathrm{NSs}}(t^{*} = t_{\mathrm{spk}}) = A(-B^{5} + B^{4} - B^{3} + B^{2} - B + 1)$$

$(2nt_{\mathrm{spk}}, (2n + 1)t_{\mathrm{spk}})$

$$t^{*} = t - 2nt_{\mathrm{spk}}$$

$$v_{\mathrm{NSs}}(t^{*} = t_{\mathrm{spk}}) = A(-B^{2n+1} + B^{2n} - B^{2n-1} + \cdots + 1)$$

Let $v_{\mathrm{NSs}}(n) = v_{\mathrm{NSs}}((2n + 1)t_{\mathrm{spk}})$

$$i_{\mathrm{ds}}(n) = \frac{v_{con}}{R_{\mathrm{con}}} = \frac{v_{\mathrm{ds}} - v_{\mathrm{NSs}}(n)}{R_{\mathrm{con}}} = \frac{V_{\mathrm{spk}} - v_{\mathrm{NSs}}(n)}{R_{\mathrm{con}}}$$

$$\Delta w(n) = \frac{i_{\mathrm{ds}}(n) - i_{\mathrm{ds}}(0)}{i_{\mathrm{ds}}(0)} = \frac{v_{\mathrm{NSs}}(n) - v_{\mathrm{NSs}}(0)}{V_{\mathrm{spk}} - v_{\mathrm{NSs}}(0)} = \frac{A(-B^{2n+1} + B^{2n} - B^{2n-1} + \cdots + B^{2})}{V_{\mathrm{spk}} - A(-B + 1)}$$

$$= \frac{AB^{2}\frac{1 - (-B)^{2n}}{1 - (-B)}}{V_{\mathrm{spk}} - A(-B + 1)}$$

$$= \frac{\frac{R_{\mathrm{NSs}}V_{\mathrm{spk}}}{R_{\mathrm{NSs}} + R_{\mathrm{con}}}e^{-\frac{R_{\mathrm{NSs}}+R_{\mathrm{con}}}{C_{\mathrm{NSs}}R_{\mathrm{con}}^{2}}2t_{\mathrm{spk}}}}{V_{\mathrm{spk}} - \frac{R_{\mathrm{NSs}}V_{\mathrm{spk}}}{R_{\mathrm{NSs}} + R_{\mathrm{con}}}\left(1 - e^{-\frac{R_{\mathrm{NSs}}+R_{\mathrm{con}}}{C_{\mathrm{NSs}}R_{\mathrm{con}}^{2}}t_{\mathrm{spk}}}\right)}\frac{1 - e^{-2\frac{R_{\mathrm{NSs}}+R_{\mathrm{con}}}{C_{\mathrm{NSs}}R_{\mathrm{con}}^{2}}t_{\mathrm{spk}}n}}{1 + e^{-\frac{R_{\mathrm{NSs}}+R_{\mathrm{con}}}{C_{\mathrm{NSs}}R_{\mathrm{con}}^{2}}t_{\mathrm{spk}}}}$$

$$= A_{1}(1 - e^{-2\frac{R_{\mathrm{NSs}}+R_{\mathrm{con}}}{C_{\mathrm{NSs}}R_{\mathrm{con}}^{2}}t_{\mathrm{spk}}n})$$

For n<0

$$v_{\mathrm{NSs}}\left(2nt_{\mathrm{spk}}\right) = -A(-B^{2n} + B^{2n-1} - \cdots + B)$$

$$(2nt_{\mathrm{spk}}, (2n+1)t_{\mathrm{spk}})$$

$$t^{*} = t - 2nt_{\mathrm{spk}}$$

$$v_{\mathrm{NSs}}(t^{*}) = -A(-B^{2n} + B^{2n-1} - \cdots + B + 1)e^{-\frac{R_{\mathrm{NSs}}+R_{\mathrm{con}}}{C_{\mathrm{NSs}}R_{\mathrm{con}}{}^{2}}t^{*}} + A$$

$$v_{\mathrm{NSs}}(-n) = v_{\mathrm{NSs}}(t^{*} = t_0) = A(B^{2n+1} - B^{2n} - \cdots - B^2) - A(B-1)$$

$$\Delta w(-n) = \frac{i_{\mathrm{ds}}(n) - i_{\mathrm{ds}}(1)}{i_{\mathrm{ds}}(1)} = \frac{v_{\mathrm{NSs}}(n) - v_{\mathrm{NSs}}(0)}{V_{\mathrm{spk}} - v_{\mathrm{NSs}}(0)} = \frac{A(B^{2n+1} - B^{2n} + B^{2n-1} - \cdots - B^2)}{V_{\mathrm{spk}} - A(-B+1)}$$

$$= -A_1(1 - e^{-2\frac{R_{\mathrm{NSs}}+R_{\mathrm{con}}}{C_{\mathrm{NSs}}R_{\mathrm{con}}{}^{2}}t_{\mathrm{spk}}n})$$

$$\Delta w(n) = -A_1(1 - e^{2\frac{R_{\mathrm{NSs}}+R_{\mathrm{con}}}{C_{\mathrm{NSs}}R_{\mathrm{con}}{}^{2}}t_{\mathrm{spk}}n})$$

$$\Delta w(n) = \begin{cases} A_1\left(1 - e^{-2\frac{R_{\mathrm{NSs}}+R_{\mathrm{con}}}{C_{\mathrm{NSs}}R_{\mathrm{con}}{}^{2}}t_{\mathrm{spk}}n}\right) & (n > 0) \\ -A_1\left(1 - e^{2\frac{R_{\mathrm{NSs}}+R_{\mathrm{con}}}{C_{\mathrm{NSs}}R_{\mathrm{con}}{}^{2}}t_{\mathrm{spk}}n}\right) & (n < 0) \end{cases}$$

$$\Delta w(n) = \begin{cases} A_1\left(1 - e^{\frac{n}{\frac{C_{\mathrm{NSs}}R_{\mathrm{con}}{}^{2}}{2t_{\mathrm{spk}}(R_{\mathrm{NSs}}+R_{\mathrm{con}})}}}\right) & (n > 0) \\ -A_1\left(1 - e^{\frac{n}{\frac{C_{\mathrm{NSs}}R_{\mathrm{con}}{}^{2}}{2t_{\mathrm{spk}}(R_{\mathrm{NSs}}+R_{\mathrm{con}})}}}\right) & (n < 0) \end{cases}$$

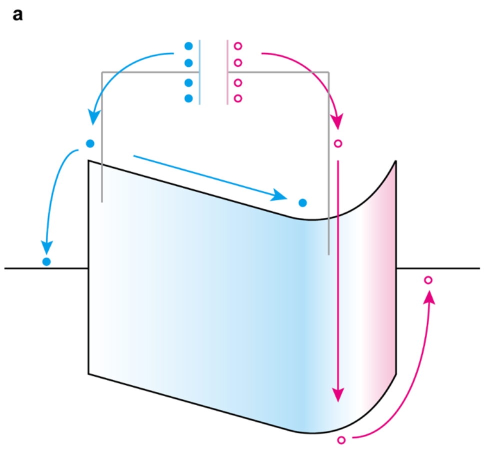

Fig. S20 Schematic figure demonstrating the effect of a gate spike. a, Before and **b,** after the gate voltage spike was applied to the device.

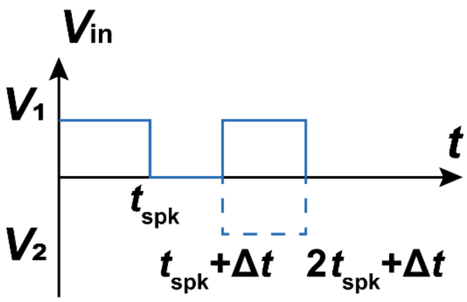

Fig. S21 Schematic figure for STDP characteristic analytical solution calculation. Two spikes applied to the device with varied interval time.

STDP is another characteristic of biological synapse and synaptic devices. In the nervous system, the synaptic weight changes when tuning the interval of two coupling spikes, which reflects the plasticity of a synapse and is known as the Hebbian learning rule.

The following is the derivation of the formula for the STDP:

$$\begin{aligned} v_{\rm ds} &= v_{\rm NSs} + v_{\rm con} \\ &= i_{R_{\rm NSs}} R_{\rm NSs} + i_{\rm ds} R_{\rm con} \\ &= i_{R_{\rm NSs}} R_{\rm NSs} + \left(i_{C_{\rm NSs}} + i_{R_{\rm NSs}}\right) R_{\rm con} \end{aligned}$$

$$\frac{C_{\rm NSs} = \dfrac{q_{C_{\rm NSs}}}{v_{\rm NSs}}}{C_{\rm NSs}\dfrac{dv_{\rm NSs}}{dt} = i_{C_{\rm NSs}}} \frac{v_{\rm NSs}}{R_{\rm NSs}}(R_{\rm NSs} + R_{\rm con}) + \frac{C_{\rm NSs} dv_{\rm NSs}}{dt} R_{\rm con}$$

$$\frac{dv_{\rm NSs}(t)}{dt} + \frac{R_{\rm NSs} + R_{\rm con}}{C_{\rm NSs} R_{\rm NSs} R_{\rm con}} v_{\rm NSs}(t) = v_{\rm ds}(t)$$

$$i_{\rm ds}(t) = \frac{v_{con}}{R_{\rm con}} = \frac{v_{\rm ds}(t) - v_{\rm NSs}}{R_{\rm con}}$$

$(0, t_{\rm spk})$

$v_{\rm ds}(\mathrm{t}) = \pm V_{\rm spk}$(± for spikes of same or opposite direction, respectively)

$$v_{\rm NSs}(t) = \tilde{c} e^{-\frac{R_{\rm NSs} + R_{\rm con}}{C_{\rm NSs} R_{\rm con}{}^2} t} \pm \frac{R_{\rm NSs} V_{\rm spk}}{R_{\rm NSs} + R_{\rm con}}$$

Boundary condition: $v_{\rm NSs}(0) = 0$

$$v_{\rm NSs}(t) = \pm \frac{R_{\rm NSs} V_{\rm spk}}{R_{\rm NSs} + R_{\rm con}} \left(1 - e^{-\frac{R_{\rm NSs} + R_{\rm con}}{C_{\rm NSs} R_{\rm con}{}^2} t}\right)$$

$$i_{\rm ds}(t) = \frac{V_0}{R_{\rm con}} \mp \frac{R_{\rm NSs} V_{\rm spk}}{R_{\rm con}(R_{\rm NSs} + R_{\rm con})} \left(1 - e^{-\frac{R_{\rm NSs} + R_{\rm con}}{C_{\rm NSs} R_{\rm con}{}^2} t}\right)$$

$$v_{\rm NSs}(t_{\rm spk}) = \pm \frac{R_{\rm NSs} V_{\rm spk}}{R_{\rm NSs} + R_{\rm con}} \left(1 - e^{-\frac{R_{\rm NSs} + R_{\rm con}}{C_{\rm NSs} R_{\rm con}{}^2} t_{\rm spk}}\right)$$

$$i_{\rm ds}(t_{\rm spk}) = \frac{V_0}{R_{\rm con}} \mp \frac{R_{\rm NSs} V_{\rm spk}}{R_{\rm con}(R_{\rm NSs} + R_{\rm con})} \left(1 - e^{-\frac{R_{\rm NSs} + R_{\rm con}}{C_{\rm NSs} R_{\rm con}{}^2} t_{\rm spk}}\right)$$

$(t_{\rm spk}, t_{\rm spk} + \Delta t)$

$v_{\rm ds}(\mathrm{t}) = 0$

Let: $t^* = t - t_{\rm spk}$

$$v_{\mathrm{NSs}}(t)=\tilde{c}e^{-\frac{R_{\mathrm{NSs}}+R_{\mathrm{con}}}{C_{\mathrm{NSs}}R_{\mathrm{con}}{}^2}t}$$

Boundary condition: $v_{\mathrm{NSs}}(t^*=0)=\pm\frac{R_{\mathrm{NSs}}V_{\mathrm{spk}}}{R_{\mathrm{NSs}}+R_{\mathrm{con}}}\left(1-e^{-\frac{R_{\mathrm{NSs}}+R_{\mathrm{con}}}{C_{\mathrm{NSs}}R_{\mathrm{con}}{}^2}t_{\mathrm{spk}}}\right)$

$$v_{\mathrm{NSs}}(t^*)=\pm\frac{R_{\mathrm{NSs}}V_{\mathrm{spk}}}{R_{\mathrm{NSs}}+R_{\mathrm{con}}}\left(1-e^{-\frac{R_{\mathrm{NSs}}+R_{\mathrm{con}}}{C_{\mathrm{NSs}}R_{\mathrm{con}}{}^2}t_{\mathrm{spk}}}\right)e^{-\frac{R_{\mathrm{NSs}}+R_{\mathrm{con}}}{C_{\mathrm{NSs}}R_{\mathrm{con}}{}^2}t^*}$$

$$i_{\mathrm{ds}}(t^*)=\pm\frac{R_{\mathrm{NSs}}V_{\mathrm{spk}}}{R_{\mathrm{con}}(R_{\mathrm{NSs}}+R_{\mathrm{con}})}\left(e^{-\frac{R_{\mathrm{NSs}}+R_{\mathrm{con}}}{C_{\mathrm{NSs}}R_{\mathrm{con}}{}^2}t_{\mathrm{spk}}}-1\right)e^{-\frac{R_{\mathrm{NSs}}+R_{\mathrm{con}}}{C_{\mathrm{NSs}}R_{\mathrm{con}}{}^2}t^*}$$

$$v_{\mathrm{NSs}}(t^*=\Delta t)=\pm\frac{R_{\mathrm{NSs}}V_{\mathrm{spk}}}{R_{\mathrm{NSs}}+R_{\mathrm{con}}}\left(1-e^{-\frac{R_{\mathrm{NSs}}+R_{\mathrm{con}}}{C_{\mathrm{NSs}}R_{\mathrm{con}}{}^2}t_{\mathrm{spk}}}\right)e^{-\frac{R_{\mathrm{NSs}}+R_{\mathrm{con}}}{C_{\mathrm{NSs}}R_{\mathrm{con}}{}^2}\Delta t}$$

$(t_{\mathrm{spk}}+\Delta t, 2t_{\mathrm{spk}}+\Delta t)$

$v_{\mathrm{ds}}(\mathrm{t})=\mathrm{V}_{\mathrm{spk}}$

Let: $t^*=t-(t_{\mathrm{spk}}+\Delta t)$

$$v_{\mathrm{NSs}}(t^*)=\tilde{c}e^{-\frac{R_{\mathrm{NSs}}+R_{\mathrm{con}}}{C_{\mathrm{NSs}}R_{\mathrm{con}}{}^2}t^*}+\frac{R_{\mathrm{NSs}}V_{\mathrm{spk}}}{R_{\mathrm{NSs}}+R_{\mathrm{con}}}$$

$$v_{\mathrm{NSs}}(t^*)=\pm\frac{R_{\mathrm{NSs}}V_{\mathrm{spk}}}{R_{\mathrm{NSs}}+R_{\mathrm{con}}}\left(1-e^{-\frac{R_{\mathrm{NSs}}+R_{\mathrm{con}}}{C_{\mathrm{NSs}}R_{\mathrm{con}}{}^2}t_{\mathrm{spk}}}\right)e^{-\frac{R_{\mathrm{NSs}}+R_{\mathrm{con}}}{C_{\mathrm{NSs}}R_{\mathrm{con}}{}^2}\Delta t}e^{-\frac{R_{\mathrm{NSs}}+R_{\mathrm{con}}}{C_{\mathrm{NSs}}R_{\mathrm{con}}{}^2}t^*}+\frac{R_{\mathrm{NSs}}V_{\mathrm{spk}}}{R_{\mathrm{NSs}}+R_{\mathrm{con}}}\left(1-e^{-\frac{R_{\mathrm{NSs}}+R_{\mathrm{con}}}{C_{\mathrm{NSs}}R_{\mathrm{con}}{}^2}t^*}\right)$$

$$i_{\mathrm{ds}}(t^*)=\frac{V_{\mathrm{spk}}}{R_{\mathrm{con}}}-\frac{R_{\mathrm{NSs}}V_{\mathrm{spk}}}{R_{\mathrm{con}}(R_{\mathrm{NSs}}+R_{\mathrm{con}})}\left(1-e^{-\frac{R_{\mathrm{NSs}}+R_{\mathrm{con}}}{C_{\mathrm{NSs}}R_{\mathrm{con}}{}^2}t^*}\right)\mp\frac{R_{\mathrm{NSs}}V_{\mathrm{spk}}}{R_{\mathrm{con}}(R_{\mathrm{NSs}}+R_{\mathrm{con}})}\left(1-e^{-\frac{R_{\mathrm{NSs}}+R_{\mathrm{con}}}{C_{\mathrm{NSs}}R_{\mathrm{con}}{}^2}t_{\mathrm{spk}}}\right)e^{-\frac{R_{\mathrm{NSs}}+R_{\mathrm{con}}}{C_{\mathrm{NSs}}R_{\mathrm{con}}{}^2}\Delta t}e^{-\frac{R_{\mathrm{NSs}}+R_{\mathrm{con}}}{C_{\mathrm{NSs}}R_{\mathrm{con}}{}^2}t^*}$$

$$i_{\mathrm{ds}}(t^*=t_{\mathrm{spk}})=\frac{V_0}{R_{\mathrm{con}}}-\frac{R_{\mathrm{NSs}}V_{\mathrm{spk}}}{R_{\mathrm{con}}(R_{\mathrm{NSs}}+R_{\mathrm{con}})}\left(1-e^{-\frac{R_{\mathrm{NSs}}+R_{\mathrm{con}}}{C_{\mathrm{NSs}}R_{\mathrm{con}}{}^2}t_{\mathrm{spk}}}\right)\mp\frac{R_{\mathrm{NSs}}V_{\mathrm{spk}}}{R_{\mathrm{con}}(R_{\mathrm{NSs}}+R_{\mathrm{con}})}\left(1-e^{-\frac{R_{\mathrm{NSs}}+R_{\mathrm{con}}}{C_{\mathrm{NSs}}R_{\mathrm{con}}{}^2}t_{\mathrm{spk}}}\right)e^{-\frac{R_{\mathrm{NSs}}+R_{\mathrm{con}}}{C_{\mathrm{NSs}}R_{\mathrm{con}}{}^2}\Delta t}e^{-\frac{R_{\mathrm{NSs}}+R_{\mathrm{con}}}{C_{\mathrm{NSs}}R_{\mathrm{con}}{}^2}t^*}$$

$$\Delta w(\Delta t)=\frac{i_{\mathrm{ds}}(2t_{\mathrm{spk}}+\Delta t)-i_{\mathrm{ds}}(t_{\mathrm{spk}})}{i_{\mathrm{ds}}(t_{\mathrm{spk}})}=\frac{\mp\frac{R_{NSs}V_{spk}}{R_{con}(R_{NSs}+R_{con})}\left(1-e^{-\frac{R_{NSs}+R_{con}}{C_{NSs}R_{con}{}^2}t_{spk}}\right)e^{-\frac{R_{NSs}+R_{con}}{C_{NSs}R_{con}{}^2}\Delta t}e^{-\frac{R_{NSs}+R_{con}}{C_{NSs}R_{con}{}^2}t^*}}{\frac{V_{\mathrm{spk}}}{R_{\mathrm{con}}}-\frac{R_{\mathrm{NSs}}V_{\mathrm{spk}}}{R_{\mathrm{con}}(R_{\mathrm{NSs}}+R_{\mathrm{con}})}\left(1-e^{-\frac{R_{\mathrm{NSs}}+R_{\mathrm{con}}}{C_{\mathrm{NSs}}R_{\mathrm{con}}{}^2}t_{\mathrm{spk}}}\right)}$$

$$=\mp(1-\frac{V_{\mathrm{spk}}}{V_{\mathrm{spk}}-\frac{R_{\mathrm{NSs}}V_{\mathrm{spk}}}{R_{\mathrm{NSs}}+R_{\mathrm{con}}}\left(1-e^{-\frac{R_{\mathrm{NSs}}+R_{\mathrm{con}}}{C_{\mathrm{NSs}}R_{\mathrm{con}}{}^2}t_{\mathrm{spk}}}\right)})e^{-\frac{R_{\mathrm{NSs}}+R_{\mathrm{con}}}{C_{\mathrm{NSs}}R_{\mathrm{con}}{}^2}\Delta t}=\mp A_2e^{-\frac{R_{\mathrm{NSs}}+R_{\mathrm{con}}}{C_{\mathrm{NSs}}R_{\mathrm{con}}{}^2}\Delta t}$$

$$(\Delta t>0)$$

$$\Delta w(\Delta t)=\begin{cases}-A_2e^{-\frac{R_{\mathrm{NSs}}+R_{\mathrm{con}}}{C_{\mathrm{NSs}}R_{\mathrm{con}}{}^2}\Delta t} & (\mathit{same\ direction})\\ A_2e^{\frac{R_{\mathrm{NSs}}+R_{\mathrm{con}}}{C_{\mathrm{NSs}}R_{\mathrm{con}}{}^2}\Delta t} & (\mathit{opposite\ direction})\end{cases}$$

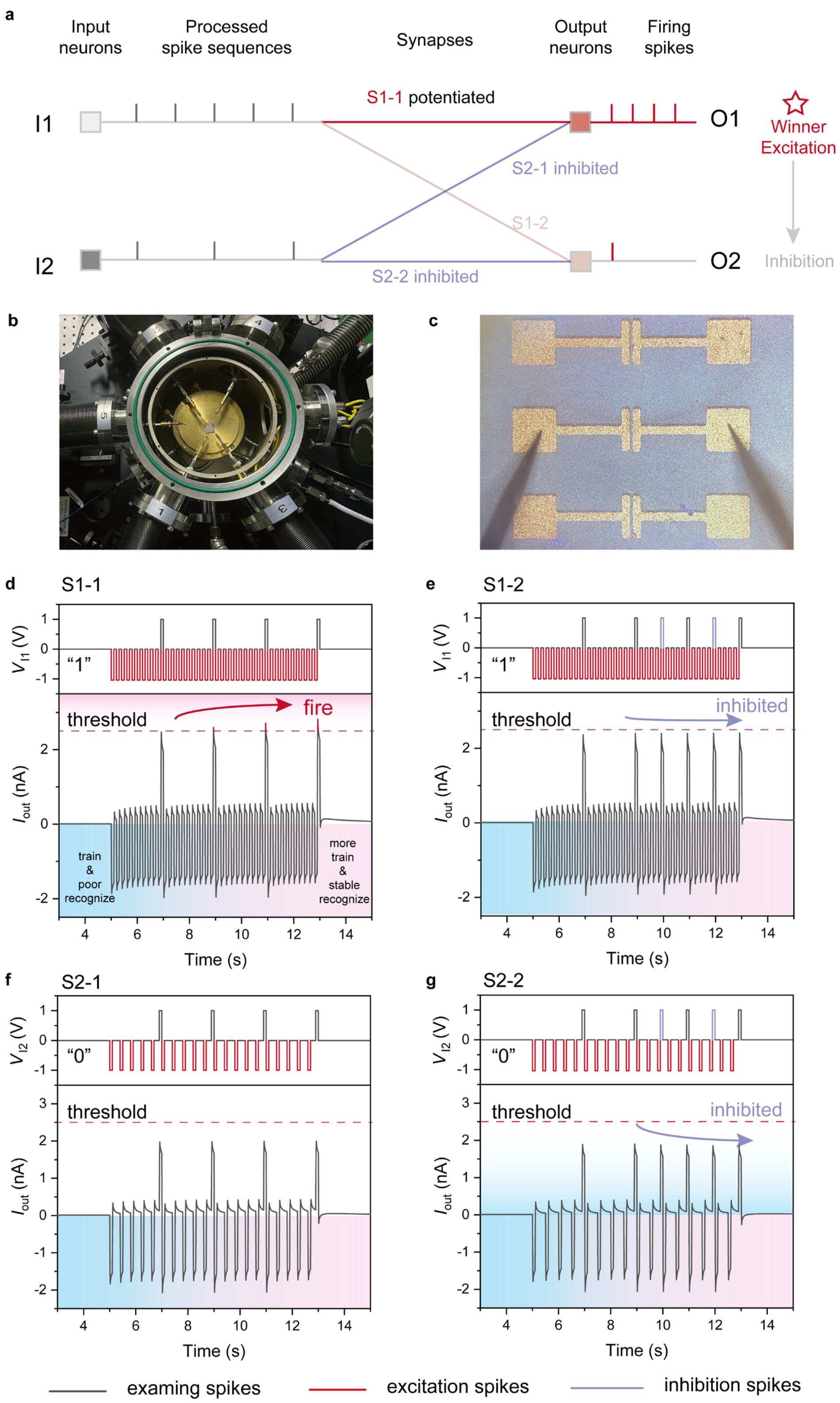

Fig. S22 The proof of concept demonstration of devices. a, The schematic figure of the simplest 2*2 classification network. **b,** The measurement setup and **c,** the optical microscopic figure of the device for synapse. **d-g,** The output current figure of the S1-1, S1-2, S2-1, S2-2 devices, respectively. The red spikes correspond to the excitation spike, the blue ones correspond to the inhibition spikes, and the black ones correspond to the examineing spikes.

We included a proof of concept demonstration of the actual device application, to verify the feasibility of the construction of scalable neuromorphic networks. We used 4 devices, which served as synapses that connected the inputs and outputs, to build the 2-input-and-2-output binary classification network. This network is the simplest classification network while sharing the same operation principle as the network we put forward in the manuscript for digit classification.

To illustrate the working mechanism, the two inputs and two outputs are labeled as I1, I2, and O1, O2, respectively. The synaptic devices connecting I_i and O_j are labeled as Si-j, e.g. the device connecting I1 and O1 is S1-1. The network was used to distinguish two opposite sequences, i.e. "I1 = 0, I2 = 1" ("0, 1") and "I1 = 1, I2 = 0" ("1, 0") for the two corresponding inputs, respectively. In this case, "0" and "1" are encoded into higher-frequency and lower-frequency spikes, respectively. And we anticipated those two sequences resulted in different outputs.

We applied the second quadrant operation pattern of the device, to demonstrate the functionality of the network. The negative voltage pulses were applied to increase the synaptic weight as excitation spikes, and the positive pulses were applied to examine if the output current of the device was over the threshold to emit an output signal as examine spikes or decrease the synaptic weight as inhabitation spikes.

In the case of the input sequence of "1, 0", i.e., I1 emitted high-frequency spikes while I2 emitted low-frequency spikes. Upon the examining pulses, we found S1-1 has the highest increasing synaptic current due to the residual weight differences or moderate device-to-device differences. In this case, the S1-1 reached the threshold (2.5 nA, labeled as red dash lines) firstly and fired O1, while O2 was inhibited according to the winner-take-all strategy. Afterward, the additional inhibition positive pulses were added to the synapse linked to O2 (S1-2 and S2-2). The inhibition pulses made the synaptic weight of S1-2 lower than S1-1 and even unlikely to reach the threshold. As for S2-1 and S2-2, the low-frequency input signal was insufficient to excite the device. Therefore, only S1-1 was enhanced and the connection between I1 and O1 continued to be enhanced, while other synaptic devices were inhibited. The O1 was then assigned to the sequences "1, 0".

For the opposite input sequence of "0, 1", i.e. the I2 emitted high-frequency spikes while I1 emitted low-frequency spikes, the opposite result could be expected. While occasionally fault classification occurred, i.e. high frequency of I1 and I2 were all related to O1. This could be due to the lack of redundancy of output neurons. Similar to the digital classification network with 300 output neurons vs 10 digits mentioned in the manuscript, the redundancy of output neurons took advantage of the device-to-device differences. With redundancy of the devices, a better-connected device can reach the threshold easier, and result in the correct classification.

Notice that, our device-based network used STDP algorithm, which was trained continuously while doing the recognition at the same time. For evaluation of the training process of this SNN, we deem it to be completed when the network is stable, and almost all the inputs can be separated into several fixed output categories without further changing as the training time prolongs. Further, for the recognition, the synapse weights can still be modified when receiving stimulus. The devices reached the stable state after one iteration of training, as the demonstrative inputs are comparably simple.

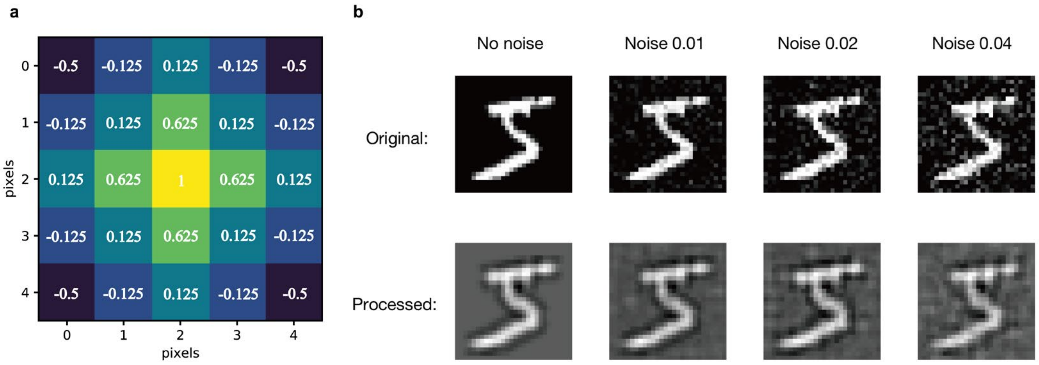

Fig. S23 Image preprocessing. a, Receptive field convolutional kernel[3]. **b,** The comparison of the original and the processed images after applying a convolutional kernel with noise variances of 0.01, 0.02, and 0.04.

Supplementary References

1. Liu, J., Yang, Y., Lyu, P., Nachtigall, P. & Xu, Y. Few-Layer Silicene Nanosheets with Superior Lithium-Storage Properties. *Adv. Mater.* **30**, 1–7 (2018).
2. Kim, M. *et al.* Zero-static power radio-frequency switches based on MoS2 atomristors. *Nat. Commun.* **9**, 1–7 (2018).
3. Iakymchuk, T., Rosado-Muñoz, A., Guerrero-Martínez, J. F., Bataller-Mompeán, M. & Francés-Víllora, J. V. Simplified spiking neural network architecture and STDP learning algorithm applied to image classification. *Eurasip J. Image Video Process.* **2015**, (2015).
